# Aurora-A condensation mediated by BuGZ aids its mitotic centrosome functions

**DOI:** 10.1016/j.isci.2024.109785

**Published:** 2024-04-18

**Authors:** Hui Zheng, Qiaoqiao Zhang, Xing Liu, Fan Shi, Fengrui Yang, Shengqi Xiang, Hao Jiang

**Affiliations:** 1 Laboratory for Aging and Cancer Research, Frontiers Science Center Disease-related Molecular Network, State Key Laboratory of Respiratory Health and Multimorbidity and National Clinical Research Center for Geriatrics, West China Hospital, Sichuan University, Chengdu 610041, Sichuan, China; 2 MOE Key Laboratory for Membraneless Organelles and Cellular Dynamics, Hefei National Center for Cross-disciplinary Sciences, University of Science & Technology of China, School of Life Sciences, Hefei, China; 3 MOE Key Lab for Cellular Dynamics, School of Life Sciences, Division of Life Sciences and Medicine, University of Science and Technology of China, Hefei 230027, China; 4 Key Laboratory of Gene Engineering of the Ministry of Education, State Key Laboratory of Biocontrol, School of Life Sciences, Sun Yat-sen University, Guangzhou 510275, Guangdong, China

**Keywords:** Cell biology, Functional aspects of cell biology, Biophysics

## Abstract

Centrosomes composed of centrioles and the pericentriolar material (PCM), serve as the platform for microtubule polymerization during mitosis. Despite some centriole and PCM proteins have been reported to utilize liquid-liquid phase separation (LLPS) to perform their mitotic functions, whether and how centrosomal kinases exert the coacervation in mitosis is still unknown. Here we reveal that Aurora-A, one key centrosomal kinase in regulating centrosome formation and functions, undergoes phase separation *in vitro* or in centrosomes from prophase, mediated by the conserved positive-charged residues inside its intrinsic disordered region (IDR) and the intramolecular interaction between its N- and C-terminus. Aurora-A condensation affects centrosome maturation, separation, initial spindle formation from the spindle pole and its kinase activity. Moreover, BuGZ interacts with Aurora-A to enhance its LLPS and centrosome functions. Thus, we propose that Aurora-A collaborates with BuGZ to exhibit the property of LLPS in centrosomes to control its centrosome-dependent functions from prophase.

## Highlights

• Aurora-A undergoes condensation *in vitro* or in mitotic centrosome from prophase
• Aurora-A coacervation is mediated by electrostatic and intramolecular interaction
• Aurora-A phase separation is required for its mitotic centrosome functions
• Aurora-A condensation mediated by BuGZ enhances its mitotic centrosome functions

## Graphical abstract

**Figure:**
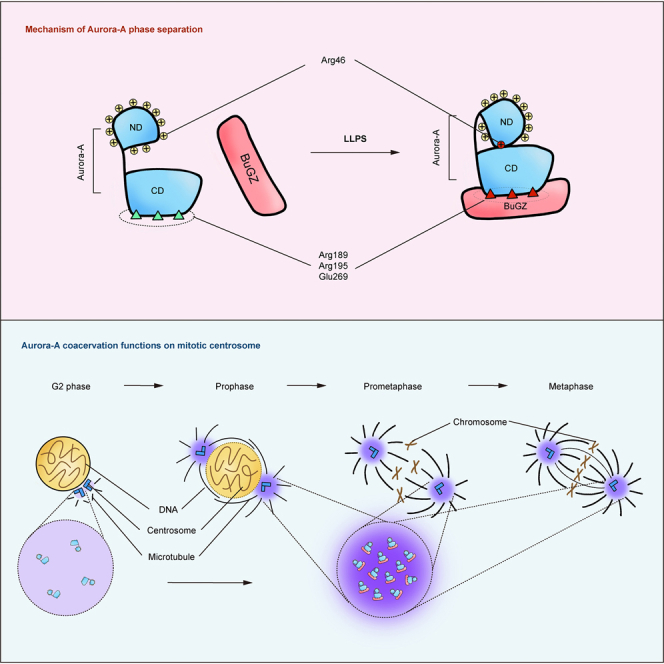

## Introduction

One pair of centrioles comprised of 9-fold triplet microtubules and scaffold proteins, and coated with external pericentriolar material (PCM), constitute the centrosome serving as the cellular microtubule-organizing center (MTOC).^1^^,^^2^^,^^3^^,^^4^^,^^5^^,^^6^ During interphase, the centrosome generates microtubules (MTs) to recruit motors and cargoes, promotes cilia assembly, establishes and maintains cellular polarity, and fulfills preparations at centrosomes for mitotic entry.^1^^,^^2^^,^^7^^,^^8^ During mitosis, the centrosome accomplishes a “maturation” process at prophase featured with PCM expansion to increase its ability of MT formation.^3^ The “maturation” process is initiated and controlled by Aurora-A-PLK1 cascade and their substantial PCM targets including Cep192, PCNT, Cep215, TACC3 and γ-tubulin.^9^^,^^10^^,^^11^^,^^12^^,^^13^ Additionally, centrosomes utilize separation to locate in the proper position to form the bipolar spindle. The separation of centrosomes starts with dissolving the “centrosome linker” composed of C-Nap-1 and rootletin at the late G2 phase, followed by dividing the centrosomes apart via the centrosomal MTs and protein motors including Eg5, dynein, and Kif15 at prophase.^14^^,^^15^^,^^16^^,^^17^ Furthermore, the centrosome modulates chromosome alignment based on its effects on spindle assembly, orientation and position, which is governed by Aurora-A, PLK1and Gαi/LGN/NuMA complex.^2^^,^^8^^,^^16^

Studying the roles and mechanisms of biological liquid-liquid phase separation (LLPS) resembling intracellular compartmentations in various physiological and pathological processes has been fueled by visualizing and disrupting the protein phase separation *in vivo* and *in vitro*. LLPS has been demonstrated to exist at different structures of the spindle during the cell division, including the spindle pole, spindle, chromosome and its associated structures.^18^^,^^19^^,^^20^^,^^21^^,^^22^^,^^23^^,^^24^ The centrosome localizing on the spindle pole and working as one of the most important mitotic subcellular structures, has been supposed to behave as a phase-separated multimolecular condensate complex.^21^^,^^25^^,^^26^ Centriolar proteins PLK4, Cep152 and Cep63 undergo phase separation to promote centriole biogenesis and self-organization.^27^^,^^28^ Among PCM proteins, SPD-5 (Cep215 homolog), PCNT, TACC3 and TPX2 have been proven to exploit the behavior of LLPS to accelerate the spindle assembly.^18^^,^^19^^,^^29^^,^^30^ Despite PLK4 acting as the centriolar kinase to exert the condensation property through its auto-phosphorylated noncatalytic cryptic polo-box (CPB) to regulate centriole duplication at G1/S phase,^27^ whether any centrosomal kinase shows the behavior of condensation, especially during mitosis and whether the coacervation regulates its mitotic centrosomal functions are still needed to decipher.

Aurora-A, the prominent centrosomal kinase for cell division, performs its centrosome-related functions during mitosis by facilitating the G2/M phase transition, promoting the centrosome maturation and separation, and initiating the MT polymerization.^6^^,^^9^^,^^11^^,^^12^^,^^31^^,^^32^^,^^33^^,^^34^ Beyond its centrosome-related roles, Aurora-A is also located in the spindle and centromere, participating in other mitotic processes, including proper Kinetochore-MT connection, MT assembly along the central spindle, establishment of spindle assembly checkpoint (SAC), and chromosome segregation.^33^^,^^35^^,^^36^^,^^37^^,^^38^^,^^39^^,^^40^^,^^41^^,^^42^ Here, we find the crucial mitotic centrosomal kinase Aurora-A, which contains intrinsic disordered region (IDR) in its N-terminal, exhibits the property of phase separation in mitotic centrosome from prophase or *in vitro*. This phase separation property mediated by the electrostatic interaction between the IDR and catalytic domain within Aurora-A and its interaction with BuGZ, promotes the centrosomal localization of Aurora-A and its functions in centrosome maturation, separation, and centrosomal MT assembly, but not the G2/M transition, which indicates that LLPS is involved in the mitotic centrosome-related kinase functions.

## Results

### Aurora-A exhibits phase separation both *in vitro* and in mitotic centrosomes

**Figure 1:**
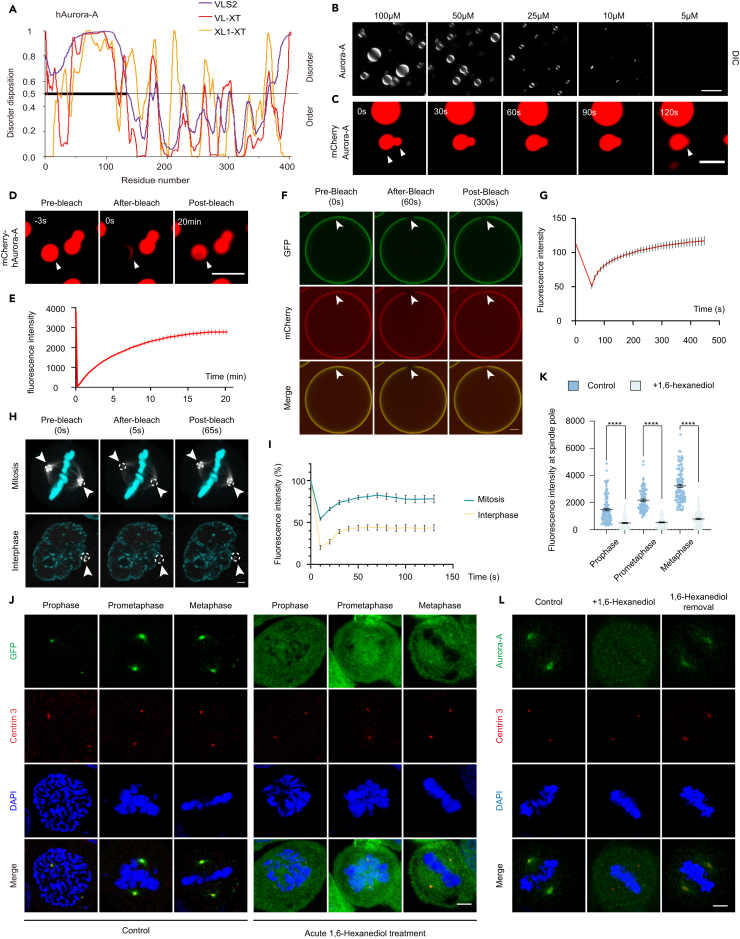
Aurora-A exhibits phase separation both *in vitro* and in mitotic centrosomes (A) Sequence features of hAurora-A predicted by PONDOR. The line at 0.5 (y axis) is the cutoff for the disorder (>0.5) and order (<0.5) predictions. VLS2, VL-XT, and XL1-XT are predictors for disordered dispositions. (B) His-hAurora-A forms concentration-dependent droplets in PBS by adding 10% PEG 6000 as visualized by DIC microscopy. Protein concentration was from 5 to 100 μM. Scale bar, 10 μm. (C) His-mCherry-Aurora-A (100 μM) undergoes droplets fusion *in vitro* as visualized by fluorescence microscopy. The white arrows indicate the fusion droplets. Scale bar, 5 μm. (D and E) Images (D) and quantification (E) of His-mCherry-Aurora-A (100 μM) fluorescence recovered after photobleaching (FRAP). The white arrows indicate the bleached droplet. 9 droplets from three independent experiments were quantified. Scale bar, 10 μm. (F and G) Images (F) and quantification (G) of FRAP on GFP-Aurora-A coated beads after adding Cherry protein (10 μM). GFP-Aurora-A coated beads were produced by pre-incubating GFP-beads (10 μL) with GFP-Aurora-A stable experessed HeLa cells (1×10^7^ cells) lysate. White arrows indicate the bleached areas. 8 of areas from three independent experiments were quantified. The fluorescence intensity remained the same before and after bleaching. Scale bar, 5 μm. (H and I) Images (H) and quantification (I) of FRAP of centrosomal GFP-Aurora-A in GFP-Aurora-A stable experessed cells. The white arrows and circles in dash line indicate the bleached areas. Cyan, Hoechst. 11 areas from mitotic centrosomes and 7 areas from interphase centrosomes were quantified. Three independent experiments were repeated. Scale bar, 5 μm. (J) Images of centrosome localization of GFP-Aurora-A in early mitosis before and after 2 min treatment with 5% 1,6- hexanediol. Centrin 3 marks centrosomes, and DAPI indicates the nucleus and chromosomes. Scale bar, 5 μm. (K) Quantifications of GFP fluorescence intensity at centrosomes with or without 1,6-hexanediol treatment (Corresponding to (J)). 50–57 total cells from three independent experiments in each experiment were measured and quantified. (L) Images of centrosome localization of endogenous Aurora-A in mitosis before and after 2 min treatment with 5% 1,6- hexanediol or hexanediol removal. Scale bar, 5 μm. Error bars indicate SEM. *Student’s t-test*: ∗∗∗∗, *p* < 0.0001.

LLPS shows multifaceted effects on the whole process of cell division,^21^^,^^22^^,^^23^ especially acting as the basis of PCM and centriole assembly.^18^^,^^27^^,^^28^^,^^30^ Nevertheless, it is still unclear whether any centrosomal kinase undergoes LLPS in mitosis and how its coacervation mediates the mitotic regulation. Interestingly, we found that Aurora-A, the key mitotic kinase located on the centrosomes, possessed a relatively conserved IDR (1-126aa) in its N-terminus by prediction, which was reckoned as the core to induce LLPS,^43^^,^^44^ whereas the most of its C-terminus, also known as catalytic domain, was more structured (Figure 1A; Figure S1A). As expected, the purified Aurora-A forms concentration-dependent droplets modulated by salt concentration and pH *in vitro* (Figure 1B; Figures S1B–S1D). Moreover, the fusion and fluorescence recovery after photo bleaching (FRAP) assays indicated the LLPS behavior of the purified Aurora-A molecules *in vitro* (Figures 1C–1E). Since our previous study unraveled that the incorporation assay could also validate the dynamics and LLPS property of candidate protein,^45^ we found that mCherry-Aurora-A but not mCherry was incorporated into the droplets formed by GFP- Aurora-A, indicating Aurora-A underwent phase separation *in vitro* (Figure S1E). Moreover, we collected the intracellular GFP-Aurora-A and its binding adaptors from mitotic HeLa cells stably expressing GFP- Aurora-A with GFP beads to mimic the mitotic centrosomes *in vivo*, followed by adding the purified mCherry tagged proteins into the beads. We found his-mCherry-Aurora-A but not his-mCherry was recruited into the surface of GFP-Aurora-A labeled beads (Figure S1F), and the fluorescence of his-mCherry-Aurora-A recovered rapidly in the GFP- Aurora-A bleached area, demonstrating the highly dynamic behavior of Aurora-A on beads (Figures 1F and 1G). To further validate the coacervation phenomenon of Aurora-A *in vivo*, we first performed FRAP assay in GFP-Aurora-A stably expressed HeLa cell, in which expressed the most similar level of GFP-Aurora-A protein to endogenous Aurora-A (Figures S1G and S1H, the middle one #2). Interestingly, the GFP-Aurora-A fluorescence recovered rapidly in mitotic but not interphase centrosomes (Figures 1H and 1I). Moreover, the recovery rate in mitotic cells was much faster than the mCherry-Aurora-A protein *in vitro* (Figures 1D and 1E; Figure S1I). Despite 1,6-hexanediol treatment lacked specificity and might lead to off-target effects,^46^ it was widely used to study the phase separation behavior of diverse candidate proteins.^47^^,^^48^^,^^49^ Here, we acutely treated cells expressing GFP-Aurora-A with 1,6-hexanediol to elucidate whether Aurora-A shows LLPS property *in vivo*. GFP-Aurora-A mainly localized at centrosomes (marked by Centrin 3) and spindle adjacent to poles in HeLa cells at the early mitotic stages (prophase, prometaphase and metaphase) under normal conditions, consistent with the previous study,^50^ whereas 1,6-hexanediol treatment destroyed the centrosome localization of Aurora-A in early mitosis (Figures 1J and 1K), but not in interphase (Figures S1J and S1K). Similarly, endogenous Aurora-A also showed the sensitivity to 1,6-hexanediol and recovered its centrosomal localization after removing hexanediol (Figure 1L). Consistently, 1,6-hexanediol treatment also suppressed the droplet formation of purified Aurora-A *in vitro* (Figure S1L). Thus, all these data manifested that Aurora-A exhibited the behavior of LLPS *in vitro* and in mitotic centrosomes.

To further determine whether the phosphorylation along with the kinase activity of Aurora-A affected its coacervation property, we purified the kinase dead mutant (D274A) and key phosphorylation mutants (T288A and S342A)^4^^,^^51^^,^^52^ (Figure S1B). We found D274A and T288A, but not S342A, slightly attenuated the droplet formation of Aurora-A *in vitro* and decreased the mitotic centrosome localization of Aurora-A when overexpressed in HeLa cells (Figures S1M–S1O), indicating the kinase activity and phosphorylation of Aurora-A might slightly influence its property of LLPS.

### Aurora-A condensation depends on the conserved positive-charged residues within the intrinsic disordered region domain and the interaction between intrinsic disordered region and catalytic domains

**Figure 2:**
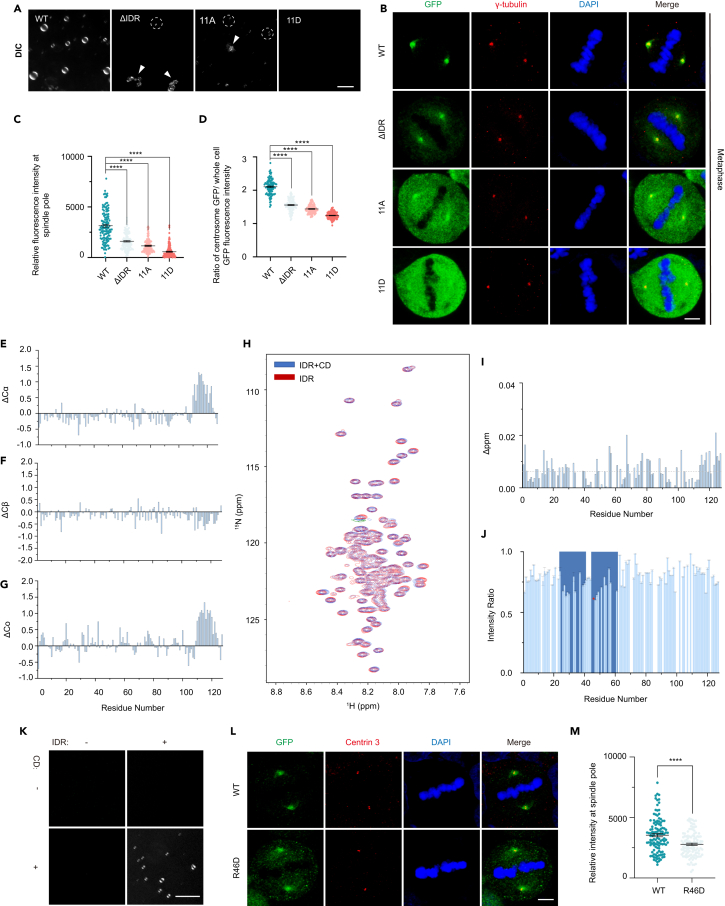
Aurora-A condensation depends on the conserved positive-charged residues within the IDR domain and the interaction between IDR and catalytic domains (A) Images of His-Aurora-WT, His-Aurora-A-ΔIDR, His-Aurora-A-11A and His-Aurora-A-11D droplet formation *in vitro* as visualized by DIC microscopy. The white arrows indicate aggregation, and the dash line circles indicate small-size droplets. Protein concentration, 50 μM. Scale bar, 10 μm. (B–D) Images (B) and Quantification (C and D) of centrosomal localization of GFP-Aurora-A-WT, GFP-Aurora-A-ΔIDR, GFP-Aurora-A-11A and GFP-Aurora-A-11D in metaphase cells. γ-tubulin marks the spindle pole. 61–65 total cells from three independent experiments in each experiment were measured and quantified. Scale bar, 5 μm. (E–G) The secondary chemical shifts of Aurora-A-IDR (1-127aa) confirmed it contains no secondary structure elements. (H) Two-dimensional ^1^H-^15^N HSQC spectra. The spectra of Aurora-A IDR in the absence (red) and in the presence (blue) of Aurora-A catalytic domain (CD, 128-403aa) (1:2 M ratio) were overlaid. (I) The chemical shift perturbations of IDR are induced by the catalytic domain (CD) at a 1:2 IDR: CD molar ratio. (J) The peak intensities changes of IDR induced by catalytic domain at a 1:2 IDR: CD molar ratio, the red star indicates Arg46. (K) Images of His-Aurora-A-IDR (5 μM) co-separated with His-Aurora-A-CD (5 μM) as visualized by DIC microscopy. Scale bar, 10 μm. (L and M) Images (K) and Quantification (L) of spindle pole localization of GFP-Aurora-A-WT and GFP-Aurora-A-R46D in metaphase cells. 57–60 total cells from three independent experiments in each experiment were measured and quantified. Scale bar, 5 μm. Error bars indicate SEM. *Student’s t-test*: ∗∗∗∗, *p* < 0.0001.

To further investigate the phase separation property of Aurora-A, we analyzed the protein sequence of its IDR among different species, within which we noticed there were 11 typically conserved positive-charged residues (lysine and arginine) whose intermolecular electrostatic interactions had also been considered as one of the LLPS modulators (Figure S1A).^53^^,^^54^ Accordingly, the purified Aurora-A depleted of IDR (ΔIDR), and the Aurora-A mutants with replacing 11 conserved positive-charged lysine and arginine with neutral-charged alanine (Aurora-A-11A) or negative-charged aspartate (Aurora-A-11D), diminished behavior of phase separation compared with wild-type Aurora-A, demonstrated by tiny droplets (indicated by dash ring) or aggregation (indicated by white arrow) (Figures S1B and Figure 2A), which proposed that LLPS of Aurora-A was mediated by the electrostatic interaction. Meanwhile, we also observed that GFP-ΔIDR, GFP-Aurora-A-11A and GFP-Aurora-A-11D, different from wild-type GFP-Aurora-A, under the condition that the total GFP fluorescence intensity of whole cells remained similar (Figure S2A), showed attenuated signal on spindle poles from prophase to metaphase (Figures 2B–2D; Figures S2B and S2C). However, all the three mutants did not affect the Aurora-A centrosome localization during interphase when treated with or without 1,6-hexanediol (Figures S1J and S1K). Thus, all these data suggested that intermolecular electrostatic interactions of these 11 conserved and positive-charged residues in the IDR mediated the LLPS of centrosomal Aurora-A during early mitosis.

We employed liquid-state NMR spectroscopy to probe the role of the interactions between the two domains of Aurora-A (IDR1-127aa) and catalytic domain (128-403aa) on its phase separation. We produced ^15^N- and ^13^C- labeled Aurora-A IDR and employed the standard triple resonance experiments to obtain backbone assignments. The secondary chemical shifts of the backbone carbon nucleus, Cα, Cβ and Co, demonstrated the region 1-110aa as a random coil, while the region 110-127aa displayed an α-helix propensity (Figures 2E–2G). In the titration experiments, we recorded ^1^H-^15^N HSQC spectra of Aurora-A IDR in the presence or absence of the unlabeled Aurora-A catalytic domain. The chemical shift perturbations were tiny (Figures 2H and 2I), indicating the binding between the IDR and catalytic domains of Aurora-A is in the slow-exchange regime. The regions 24-41aa and 45-61aa showed the most significant signal drops, which revealed that this region of IDR was involved in the interaction with the catalytic domain (Figure 2J). Furthermore, adding purified IDR domain to Aurora-A catalytic domain promoted the droplet formation (Figure 2K). Combining the sequence conservation analysis (Figure S1A) and assays with the mutant Aurora-A-11A or Aurora-A-11D (Figures 2A–2D; Figures S2A–S2C), we speculated that the Arg46 as one of the 11 conserved and positive-charged residues might dominate the interaction between the IDR and catalytic domain and the LLPS of Aurora-A. To validate this point, we created the mutant Aurora-A-R46D and found it strikingly impaired the LLPS of Aurora-A *in vitro* (Figure S2D), and diminished centrosomal localization in metaphase cells (Figures 2L and 2M). It revealed that the electrostatic interaction mediated by Arg46 was the key player in the intramolecular interaction of Aurora-A and its property of LLPS. In addition, we found that ADP also interacts with IDR, revealed by the peak shifts in the titration of ADP (Figure S2E). The elevated chemical shifts perturbation (CSP) and the intensities changes upon titration manifested the binding sites within the IDR (Figures S2F and S2G). The negatively charged ADP might modulate the charge properties of the Aurora-A IDR, thereby affecting the LLPS of the Aurora-A through the electrostatic interactions. Despite most previous studies focused on the catalytic domain rather than the IDR of Aurora-A,^55^^,^^56^ we indicated that IDR in the N-terminal of Aurora-A played a crucial role in its phase separation. In addition, our results revealed that the interaction between IDR and catalytic domains of Aurora-A mediated its LLPS. As a ligand of the catalytic domain, ADP binding to the IDR region might also modulate the LLPS behavior of Aurora-A, which links the LLPS of Aurora-A to its kinase activities and biological functions.

### The liquid-liquid phase separation of Aurora-A is not required for the G2/M phase transition

**Figure 3:**
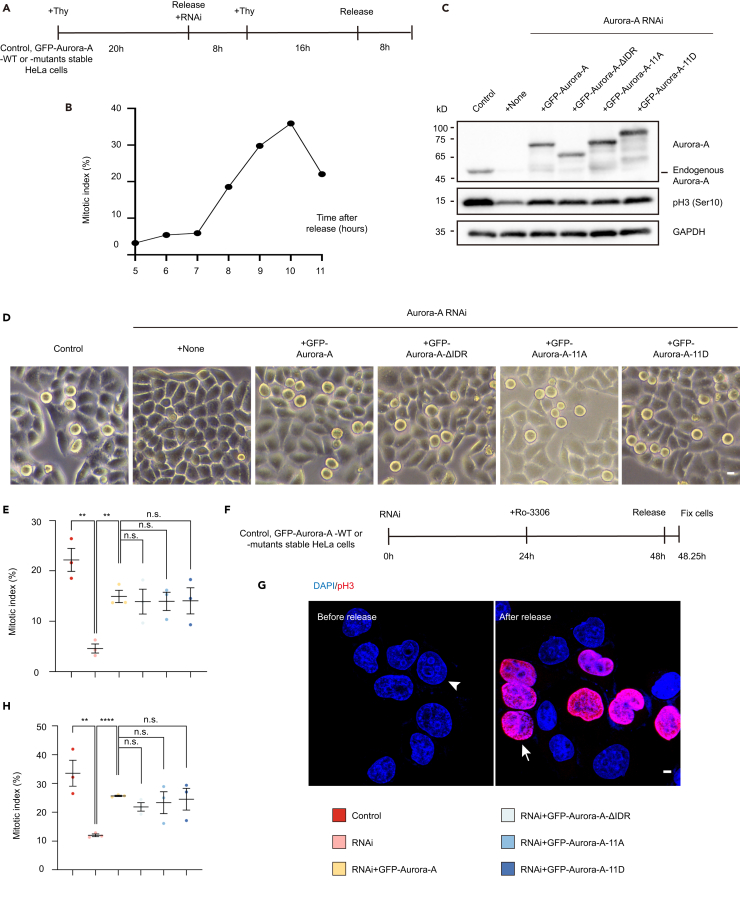
The LLPS of Aurora-A is not required for G2/M phase transition (A) and (F) Schemes of cell synchronization and RNAi treatment. (B) Mitotic index increased sharply 8 h after double thymidine block and release. (C) Cells transfected with siRNA were synchronized, collected at 8 h after double thymidine block and release, and analyzed by Western blotting using indicated antibodies. (D) Bright field Images of cells manipulated with Aurora-A RNAi and rescue. Images were shot at 8 h after double thymidine release. Scale bar, 10 μm. (E) Mitotic index corresponding to (D). Approximately 500 total cells from three independent experiments in each experiment were counted. (G) Images of cells treated with Ro-3306 block and release. pH3 marks cells in prophase. Scale bar, 10 μm. (H) Mitotic index corresponding to (F and G). Approximately 500 total cells from three independent experiments in each experiment were counted. Error bars indicate SEM. *Student*’s t test: n.s., no significance; ∗∗, *p* < 0.01; ∗∗∗∗, *p* < 0.0001.

As centrosomal Aurora-A behaved the property of phase separation from prophase, it was important to investigate whether Aurora-A condensation at the centrosome contributed to its centrosomal functions in mitosis. The centrosome localization of Aurora-A was demanded for G2/M transition, centrosome maturation and separation, and centrosomal MT nucleation and polymerization. Among these functions, prompting mitosis entry by phosphorylating CDC25B to activate Cyclin B/CDK1 complex at late G2 phase was the initial involvement of centrosomal Aurora-A during mitosis.^4^^,^^6^^,^^11^^,^^32^ Our data showed that 1,6-hexanediol treatment for interphase cells did not dissolve the centrosomal Aurora-A (Figures S1J and S1K), implying the centrosome localization of Aurora-A in interphase might be independent of LLPS. To figure out this hypothesis, we synchronized the control and Aurora-A RNAi cells with or without overexpressing Aurora-A WT or its mutants (ΔIDR, Aurora-A-11A or Aurora-A-11D) by double thymidine block and release to study the role of Aurora-A coacervation on the G2/M transition (Figure 3A). Analysis of the percentage of mitotic cells treated by the scheme in Figure 3A exhibited a notable increased mitotic index from 8 h after the release (Figure 3B). Western blotting analyses using cells collected at 8 h after the whole treatment and phosphorylated histone 3 (pH3) as the mitosis marker, proposed that defect of mitotic entry caused by Aurora-A depletion could be rescued either by wild-type Aurora-A or by its condensation deficient mutants (ΔIDR, Aurora-A-11A or Aurora-A-11D) (Figure 3C). To double confirm this conclusion, mitotic indexes were represented by counting round mitotic cells under bright field, whose results indicated Aurora-A mutants, including ΔIDR, Aurora-A-11A and Aurora-A-11D, compared with Aurora-A-WT, showed no difference in remedying the G2/M transition defect with Aurora-A RNAi (Figures 3D and 3E). With treating cells with CDK1 inhibitor Ro-3306 to block them into the G2 phase and synchronizing cells into prophase by releasing,^57^^,^^58^ we also found that LLPS of Aurora-A was not involved in its function of promoting G2/M transition by calculating prophase cells with pH3 staining (Figures 3F–3H). Thus, consistent with its non-condensation behavior on centrosomes in interphase, Aurora-A coacervation did not function in the G2/M transition.

### Aurora-A condensation prompts centrosome maturation, separation and microtubule formation from the poles

**Figure 4:**
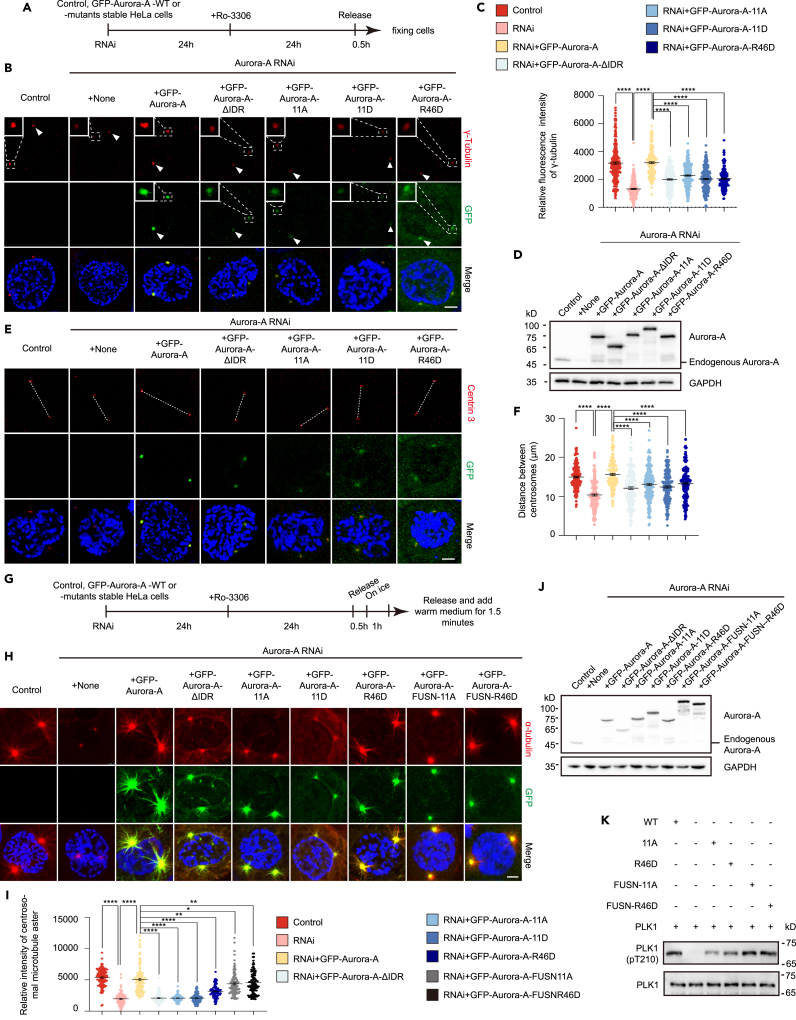
Aurora-A condensation prompts centrosome maturation, separation and MT formation from the poles (A and G) Scheme of cell synchronization and RNAi treatment. (B–F) Cells manipulated by Aurora-A RNAi and rescue treatment were stained with antibodies to γ-tubulin (B) or centrin 3 (E). Blue, DAPI. The endogenous and exogenous protein levels were indicated by Western blotting (D). The relative fluorescence intensities of γ-tubulin (C) and the distance between centrosomes (F) were measured. 85–135 total cells and 200 total cells, respectively, from three of independent experiments in each experiment, were measured and quantified for centrosome maturation and distance measurement. Scale bar, 5 μm. (H–J) Cells manipulated by Aurora-A RNAi and rescue treatment were stained with antibodies to α-tubulin (H). The relative fluorescence intensity of centrosomal α-tubulin (I) was measured. 45–50 total cells from three independent experiments in each experiment were measured and quantified. The endogenous and exogenous protein levels were indicated by Western blotting (J). Scale bar, 5 μm. (K) Purified His-Aurora-A-WT, -11A and -R46D were incubated with PLK1 to assess kinase activity *in vitro*. Error bars indicate SEM. *Student’s t test*: ∗, *p* < 0.5; ∗∗, *p* < 0.01; ∗∗∗∗, *p* < 0.0001.

Centrosomes serving as cellular MTOC was essential for spindle assembly during cell division.^8^ Centrosomes accomplished maturation at prophase, featured with PCM expansion and increase of the ability of and MT assembly.^1^^,^^3^^,^^59^ Although LLPS had been regarded as the basic requirement to drive centrosome maturation,^25^^,^^26^ the detailed mechanism was still vague. As Aurora-A ruled centrosome maturation^9^^,^^31^^,^^50^^,^^60^ and exhibited the coacervation behavior on the mitotic centrosomes in our study (Figures 1H–1L; Figures 2B and 2D), we hypothesized that the LLPS of Aurora-A might participate in its centrosome functions during mitosis. We first synchronized cells with the CDK1 inhibitor Ro-3306 block and release to prophase which indicated by condensed chromatins and intact nuclear envelope staining (Figure 4A; Figure S3A). We found that Aurora-A RNAi led to centrosome immature represented by decreased γ-tubulin signal on centrosomes when cells were released to prophase, which was rescued by GFP-Aurora-A, whereas GFP-Aurora-A-ΔIDR, GFP-Aurora-A-11A, GFP-Aurora-A-11D or GFP-Aurora-A-R46D showed attenuated effects on rescuing the centrosome localization of γ-tubulin (Figures 4B and 4C). The endogenous and exogenous protein levels of Aurora-A were indicated by western blotting (Figure 4D). Besides, both immunofluorescence and 3-dimensional reconstruct manifested that the overexpression of Aurora-A condensation deficient mutant GFP-Aurora-A-ΔIDR, GFP-Aurora-A-11A, GFP-Aurora-A-11D or GFP-Aurora-A-R46D shortened the distance between centrosomes compared with GFP-Aurora-A in Aurora-A depleted prophase cells (Figures 4E and 4F; Figure S3B), manifesting that the centrosome separation was also controlled by Aurora-A coacervation. Then, we tested whether the phase separation of Aurora-A affected the initial MT assembly from centrosomes, utilizing MT depolymerization and regrowth assay by cold treatment with cells synchronized with Ro-3306 block and release (Figure 4G). The reduced intensity of MTs emitting from centrosomes caused by Aurora-A RNAi could be largely rescued by expressing GFP-Aurora-A rather than GFP-Aurora-A-ΔIDR, GFP-Aurora-A-11A, GFP-Aurora-A-11D, or GFP-Aurora-A-R46D in prophase cells stained with α-tubulin to assess spindle formation (Figures 4H and 4I). Western blotting was employed to show endogenous and exogenous protein levels of Aurora-A (Figure 4J). As the previous report mentioned that compromised MT polymerization resulted from centrosome maturation might lead to misaligned chromosomes,^2^^,^^61^ we also checked the effects of Aurora-A LLPS on chromosome alignment and MT intensity of the spindle body. We found Aurora-A condensation deficient mutants (GFP-Aurora-A-ΔIDR, GFP-Aurora-A-11A, GFP-Aurora-A-11D and GFP-Aurora-A-R46D) showed fewer effects on counteracting the decrease of the bulk spindle intensity and the increase of mitotic cells with misaligned chromosomes caused by Aurora-A depletion, compared with GFP-Aurora-A (Figures S3D–S3F). Mitotic cells were collected by mitotic shake off and analyzed by western blotting for probing endogenous and exogenous Aurora-A levels (Figure S3G). To exclude the ascendent protion of cells with misalignment chromosomes introduced by the presence of prometaphase cell, we performed live cell imaging to reassure the conclusion (Figure S3H). As Aurora-A exerted its mitotic functions mainly through its kinase activity, we also examined whether the Aurora-A mutants deficient in undergoing LLPS diminished the kinase activity by *in vitro* kinase assay. Compared with wild-type Aurora-A, both Aurora-A-11A and Aurora-A-R46D showed attenuated kinase activity by phosphorylating the direct substrate of Aurora-A PLK1.^9^^,^^10^ Since our FRAP assays mainly showed a dynamic turnover of Aurora-A on the beads and centrosomes rather than its LLPS behavior *in vivo* (Figures 1F–1I), we also fused the FUS-IDR domain with the Aurora-A LLPS-deficient mutants, and explored the localizations and the rescue effects of the FUS-IDR fused mutants to further study the LLPS behavior of Aurora-A. By introducing the N-terminal of FUS (IDR region, 1-214aa), which is highly disordered and widely used to add into candidate LLPS-deficient mutants to study the behavior and function of the candidate condensate *in vivo* or *in vitro*,^62^^,^^63^^,^^64^ to the Aurora-A mutant 11A and R46D, both Aurora-A-FUSN-11A and Aurora-A-FUSN-R46D partially restored the phase separation of Aurora-A and rescued the deficiency in MT polymerization caused by Aurora-A RNAi and promoted the PLK1 phosphorylation, referring that the phase separation of Aurora-A contributed to its mitotic centrosome functions (Figures 4H–4K). Thus, our data suggested that the LLPS of Aurora-A promoted centrosome maturation, centrosome separation, MT assembly from centrosomes and its kinase activity.

### BuGZ enhances the property of Aurora-A coacervation *in vivo* and *in vitro*

**Figure 5:**
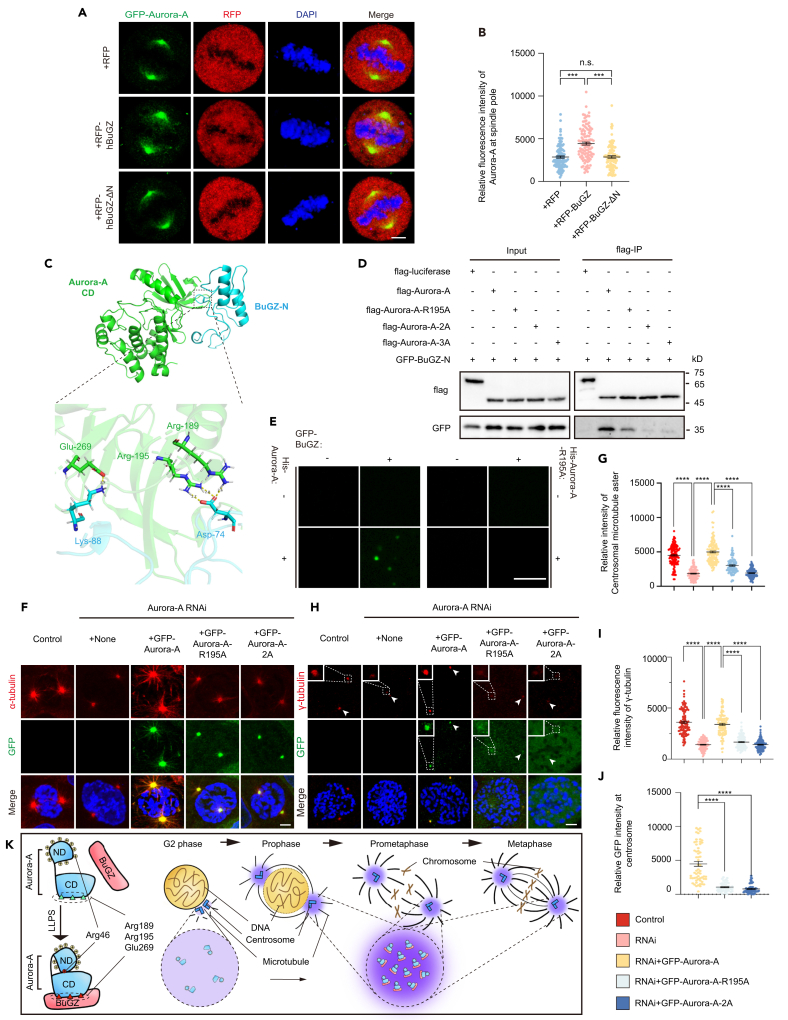
BuGZ enhances the property of Aurora-A coacervation *in vivo* and *in vitro* (A and B) Images (A) and quantification (B) of centrosomal GFP-Aurora-A in metaphase HeLa cells which transfected with RFP, RFP-BuGZ or RFP-BuGZ-ΔN. 50 total cells from three independent experiments in each experiment were measured and quantified. Scale bar, 5 μm. (C) Molecular docking sites between Aurora-A catalytic domain (Aurora-A-CD) and BuGZ-N-terminus (BuGZ-N). (D) Co-immunoprecipitation of flag-luciferase, flag-Aurora-A-WT or -mutants with GFP-BuGZ-N in HEK293T cell lysate. (E) Images of co-separation of His-Aurora-A-WT (5 μM) or -R195A (5 μM) with His-GFP-BuGZ (1 μM), visualized by fluorescence microscopy. Scale bar, 10 μm. (F and G) Cells manipulated by RNAi and rescue treatment were stained with antibodies to α-tubulin (F). Blue, DAPI. The relative fluorescence intensity of centrosomal α-tubulin (G) was measured. 46–50 total cells from three independent experiments in each experiment were measured and quantified. Scale bar,5 μm. (H–J) Cells manipulated by Aurora-A RNAi and rescue treatment were stained with antibodies to γ-tubulin (H) to indicate centrosome maturation. Blue, DAPI. The relative fluorescence intensities of γ-tubulin (I) and GFP (J) were measured. 46–60 total cells from three independent experiments in each experiment were measured and quantified. Scale bar,5 μm. (K) A model elucidating that Aurora-A condensation mediated by BuGZ aids its mitotic centrosome functions. Error bars indicate SEM. *Student’s t-test*: n.s., no significance; ∗∗∗, *p* < 0.001; ∗∗∗∗, *p* < 0.0001.

Beyond its interphase functions of mRNA splicing, synaptic vesicles (SV) cycling, and reprogramming of embryonic stem cells,^65^^,^^66^^,^^67^^,^^68^ BuGZ played vital roles on regulating proper mitotic progresses including microtubule (MT) polymerization, MT-kinetochore interaction, and SAC.^69^^,^^70^^,^^71^^,^^72^^,^^73^^,^^74^^,^^75^ Using Aurora-A beads assay with the *Xenopus* egg extract, BuGZ was not only localized at spindle body, but also enriched at spindle poles centered with xAurora-A-coated beads,^45^ which implied that BuGZ might interact with Aurora-A to contribute to centrosome-related mitotic functions. Additionally, it was clarified that xBuGZ interacted with xAurora-A catalytic domain via its zinc fingers domain in the N-terminus to enhance the kinase activity of Aurora-A.^70^ We confirmed this interaction between hAurora-A and hBuGZ by pull down assay, by which we found the purified BuGZ-N-terminus (GST-BuGZ-N, 1-92aa) directly bound to Aurora-A catalytic domain (His-Aurora-A-CD, 122-403aa) (Figure S4A). Moreover, adding GFP-BuGZ to mCherry-Aurora-A accelerated their co-condensation (Figure S3B). Overexpressing RFP-BuGZ, but not RFP or RFP-hBuGZ-ΔN, prompted the pole-localization of GFP-Aurora-A in mitotic HeLa cells (Figures 5A and 5B). Furthermore, we discovered that the interaction between Aurora-A and BuGZ in mitosis was stronger than in G2 phase (Figure S4C), and both RFP-BuGZ and GFP-Aurora-A showed increased signal on centrosomes in prophase cells than in G2 phase cells (Figures S4D–S4F), implying that the loading of BuGZ to centrosomes and the binding of BuGZ to centrosomal Aurora-A might mediate the LLPS of Aurora-A in centrosomes. Thus, BuGZ promoted Aurora-A condensation *in vitro* and in mitotic centrosomes, which might explain why Aurora-A underwent phase separation during mitosis but not interphase.

To further validate the contribution of the interaction between BuGZ and Aurora-A to Aurora-A coacervation, we proceeded with the molecular docking of Aurora-A-CD to BuGZ-N. Flexible docking was performed by using the RosettaDock and the lowest energy conformation, inferred the interaction relied on the residue Arg195, Arg189 and Glu269 within the Aurora-A catalytic domain (Figure 5C). By creating the mutants Aurora-A-R195A, Aurora-A-2A (R195A and R189A) and Aurora-A-3A (R195A, R189A and E269A), we found all three mutants attenuated their binding to BuGZ-N, analyzed by immunoprecipitation and western blotting (Figure 5D). Adding Aurora-A but not Aurora-A-R195A promoted the co-condensation with BuGZ *in vitro* (Figure 5E). Moreover, all three mutants Aurora-A-R195A, Aurora-A-2A and Aurora-A-3A, exerted weakened localizations on the centrosomes illustrated by Centrin3 (Figures S4G and S4H). Additionally, we found that both Aurora-A-R195A and Aurora-A-2A overexpression showed a diminished rescue effect on initial pole-directed spindle assembly and centrosome maturation, and weakened centrosome localization in prophase cells treated with Aurora-A RNAi, compared with wild type Aurora-A overexpression (Figures 5F–5J).

## Discussion

Extensive efforts have been devoted to understanding how the centrosomal functions of Aurora-A are exerted and proceeded during the mitotic process. By exploring the LLPS behavior of Aurora-A, we have deciphered that Aurora-A shows the specific behavior of phase separation in mitotic but not interphase centrosomes. Aurora-A condensation is modulated by 11 conserved and positive-charged residues within the IDR of its N-terminus and the cooperation between the IDR and catalytic domain mediated by Arg46 among the 11 amino acids. Besides, we uncover the principal role of Aurora-A condensation on its centrosomal functions in prophase, including centrosome maturation, separation, and the centrosome-mediated microtubule polymerization. Moreover, BuGZ, another key mitotic regulator, interacts with Aurora-A to accelerate its LLPS property and promote its mitotic functions on centrosomes. The key residues Arg195, Arg189 and Glu269 within the catalytic domain of Aurora-A, modulate the interaction with BuGZ, further adjusting its phase separation and centrosomal functions (Figure 5K). Therefore, our study opens the door to exploring the roles of Aurora-A coacervation on other Aurora-A-related biological processes beyond its centrosome functions in prophase, especially during mitosis. Our study also provides an new angle to study the involvement of LLPS in centrosome assembly and orientation. Additionally, this broadened understanding of the roles of LLPS during mitosis, especially for the key centrosomal kinase condensation, may facilitate the selection of other mitotic kinases exerting LLPS property in the effort of developing new cognitions for the relationship between phase separation and mitosis.

In our study, we adopted FRAP assays, 1,6-Hexanediol treatment and created LLPS-deficient mutants of Aurora-A fused with or without FUS-IDR region, to verify the Aurora-A condensation *in vitro* and in mitotic centrosomes. Even though some of the assay strategies were not perfect due to the methodology limits such as FRAP assays and 1,6-Hexanediol treatment, the combined data showed the condensation property of Aurora-A in spindle poles from prophase and revealed the mediation of Aurora-A LLPS in mitotic centrosome functions. Considering these methodology limits, we have been working on setting up the correlative light and electron microscopy (CLEM) platform, which has been widely performed recently in many LLPS investigations to confirm the phase separation property of candidate proteins *in vivo*.^76^^,^^77^^,^^78^ It might be a powerful and effective tool to explore Aurora-A LLPS in depth.

There might be two hypotheses to explain how the FUS IDR domain restored the condensation capacity of Aurora-A: 1) Since FUS IDR carries a negative charge while Aurora-A catalytic domain carries a positive charge, FUS IDR might interact with Aurora-A catalytic domain via electrostatic interaction, which in turn accelerates Aurora-A LLPS; 2) Due to the highly disordered structure and substantial pro-LLPS ability of FUS IDR, it might restore the Aurora-A condensation by itself through the inter- and/or intra-molecular interactions within/between the FUS IDR.

In this study, we proposed that Aurora-A underwent LLPS through conserved IDR within its N-terminus when entering mitosis (Figures 1H and 1I; Figures S1J and S1K). Previous reports have demonstrated that the N-terminal domain of Aurora-A exhibits E3 ubiquitin ligase activity.^79^^,^^80^ The E3 ligase activity of Aurora A is critical for regulating centrosomal OLA1 and centrosome amplification in G2 phase.^81^ Whether the N-terminal domain of Aurora-A promotes or suppresses the ligase activity or undergoes a transformation in its identity for spatiotemporal regulation by phase separation in mitosis still needs further exploration.

The separation of centrosomes is controlled by multifaceted motors, including Eg5, Dynein, kif15 MACK, kif18b et al. Eg5 is the most studied one, whose depletion induced obviously monopolar spindle in mitosis.^15^ Eg5 spread in the cytoplasm in interphase, while shifted onto spindle and centrosomes during cell division.^82^ In mitosis, Eg5 localized on anti-parallel MTs and generated antiparallel force to push centrosomes apart in G2-early prophase.^83^ Eg5 was reported to be phosphorylated by Aurora-A,^17^ although there was no evidence to supported that this process promotes centrosomes separation. Dynein is another necessary regulator to mediate centrosomes separation.^84^ Dynein localized around nuclear envelope (NE), produced force to antagonize Eg5 via Lis/CLIP-170 complex.^85^ Kif-15 also played an important role in maintaining inter-centrosomes distance. Distinct to Eg5, kif-15 localized in K-MT after the bipolar spindle formation to assist Eg5 to stabilize the spindle. In such cells that Eg5 did not play dominant role, kif-15 was recruited to non-K-MTs to replace the function of Eg5. Phosphorylated by Aurora-A was contributed this shift.^14^ Besides, MCAK- or kif18b-dependent MT depolymerization was also mentioned to contribute bipolar spindle formation when Eg5 was inhibited.^14^ MTs and their associated motors have been regarded as the main driver of centrosome separation, however, the role of actin dynamics in this process has been gradually noticed.^86^^,^^87^ Recently, prophase-specific perinuclear actin has been referred to as a coordinate of centrosome separation, in which the linker of nucleoskeleton and cytoskeleton (LINC) complex with perinuclear actin filaments is the crucial factor.^88^ Among the mentioned factors, Eg5 and kif15 were the definitely reported substrates of Aurora-A, but in this research, we did not investigate the specific mechanism for Aurora-A condensate to regulate centrosomes separation. Whether Aurora-A coacervation was involved in mediating the level or localization of these factors is still worthy further exploration.

The kinase activity of Aurora-A modulates the entire process of centrosome maturation, separation and initial spindle formation during prophase.^89^^,^^90^^,^^91^ Both auto-phosphorylation and adaptor mediation including TPX2, CEP192 and INCENP, modulated the activation of Aurora-A,^9^^,^^89^^,^^92^^,^^93^^,^^94^^,^^95^^,^^96^ Interestingly, we observed that Aurora-A condensation enhanced the kinase activity of Aurora-A (Figure 4K). Thus, Aurora-A condensation may be involved in auto-phosphorylation or/and adaptor mediation to regulate Aurora-A activation *in vivo*. First, Aurora-A coacervation may enhance its dimerization and autophosphorylation independent on other adaptors by raising its subcellular protein concentration on centrosomes. Second, Aurora-A exhibits the behavior of LLPS on centrosomes from prophase, while centrosome-located Cep192 accelerates the dimerization of Aurora-A and acts as the prophase regulator of Aurora-A to promote centrosome maturation and bipolar spindle assembly.^34^^,^^92^ Thus, centrosome-located Aurora-A condensate may recruit needful amount of Cep192 to the centrosomes to perform Aurora-A activation in turn. Third, beyond the centrosomes, Aurora-A is also located in the spindle and kinetochore mediated by TPX2 and INCENP in mitosis.^51^^,^^94^^,^^97^^,^^98^ Meanwhile, both TPX2 and the CPC complex containing INCENP undergo LLPS *in vitro* or *in vivo*.^19^^,^^20^ Thus, Aurora-A may also show the LLPS behavior in the spindle and/or kinetochore. Furthermore, its condensation may accumulate TPX2 and INCENP in the spindle and kinetochore and increase their level to perform their respective phase separation. The coacervation of TPX2 or INCENP may in turn activate Aurora-A along the spindle. Above all, considering that diverse adaptors are involved in Aurora-A activation during mitosis, the Aurora-A LLPS may be not sufficient to support its activation.

In this study, we uncover the relationship between LLPS and the centrosome-located mitotic kinase, provide another angle to understand the role of LLPS on centrosome assembly and maturation, and discover the intracellular functions of Aurora-A condensation in centrosome during prophase.

### Limitations of the study

In this study, we reported the LLPS of Aurora-A *in vitro* and in mitotic centrosomes by employing methods of FRAP, 1,6- Hexanediol treatment and rescued assay with LLPS-deficient mutants of Aurora-A fused with or without FUS-IDR region. However, due to the high centrosome dynamics during maturation and the side effect of 1,6-Hexanediol treatment, we still need to perform extra experiments to validate the Aurora-A condensation in the further. Since the correlative light and electron microscopy (CLEM) has been widely adopted recently in many LLPS investigations to confirm the phase separation property of candidate proteins *in vivo*, it is possible to explore Aurora-A condensate deeply by performing the CLEM assay.

## STAR★Methods

### Key resources table

**Table:** 

REAGENT or RESOURCE	SOURCE	IDENTIFIER
**Antibodies**		

Aurora A	Cell Signaling Technology	Cat#91590; RRID:AB_2800171
GAPDH	Proteintech	Cat#60004-1-Ig; RRID:AB_2107436
GFP	Abcam	Cat# ab13970; RRID:AB_300798
α-tubulin	Sigma-Aldrich	Cat# T9026; RRID:AB_477593
γ-tubulin	Sigma-Aldrich	Cat# T5192; RRID:AB_261690
Centrin 3	Abcam	Cat# ab228690
pH3 (Ser10)	Millipore	Cat#06–570; RRID:AB_310177
PLK1	Proteintech	Cat#10305-1-AP; RRID:AB_2877731
PLK1(pT210)	BD Biosciences	Cat#558400; RRID:AB_647292
HRP-flag	Proteintech	Cat# HRP-66008; RRID:AB_2883835
HRP-GFP	Proteintech	Cat# HRP-66002; RRID:AB_2883834
BuGZ	Sigma-Aldrich	Cat# HPA017013; RRID:AB_1859124
HRP-His	Proteintech	Cat# HRP-66005; RRID:AB_2857904
Lamin B1	Abcam	Cat# ab229025; RRID:AB_3083735

**Bacterial and virus strains**		

DH5α	Transgen Biotech	Cat#CD201
BL21 (DE3)	Transgen Biotech	Cat# CD601
Rosetta (DE3)	Weidibio	CAT#: EC1010

**Chemicals, peptides, and recombinant proteins**		

Uni Seamless Cloning and Assembly Kit	Transgene	Cat#CU101
Q5® Site-Directed Mutagenesis Kit	New England Biolabs	Cat#E0554S
DMEM	Thermo Fisher Scientific	Cat# C11995500
Opti-MEM	Thermo Fisher Scientific	Cat#31985070
Grace’s Insect Medium, supplemented	Thermo Fisher Scientific	Cat#11605094
Grace’s Insect Medium, unsupplemented	Thermo Fisher Scientific	Cat# 11595030
FBS	Excell	Cat#FSP500
RNAiMAX	Thermo Fisher Scientific	Cat# 13778150
Lipo 3000	Thermo Fisher Scientific	Cat# L3000015
MG132	MCE	Cat#HY-13259
Thymidine	Sigma-Aldrich	Cat#T0895
Ro-3306	MCE	Cat#HY-12529
Ni-NTA Agarose	QIAGEN	Cat# 30230
Glutathione-agarose	Thermo Fisher Scientific	Cat#16101
lysis buffer	Beyotime	Cat# P0013
PMSF	Roche	Cat# 329-98-6
EDTA-free protease inhibitor cocktail	MCE	Cat# HY-K0010
Flag-agarose	Sigma-Aldrich	Cat# A2220
GFP-Trap agarose	Chromotek	Cat# gta-20

**Critical commercial assays**		

Molecular docking	Wuhan Yangene Biological Technology	N/A

**Experimental models: Cell lines**		

HeLa	ATCC	Cat# CCL-2 RRID:CVCL_0030
HEK293T	ATCC	RRID:CVCL_0063
SF-9	ATCC	Cat# CRL-1711, RRID:CVCL_0549

**Oligonucleotides**		

Primers for protein expression constructs, see Table S1	This paper	N/A
Aurora-A siRNA	GenePharma	N/A
Control siRNA	GenePharma	N/A

**Software and algorithms**		

ImageJ	ImageJ	https://imagej.nih.gov/ij/
LAS X	Leica	N/A
TopSpin	Bruker	https://www.bruker.com/en/products-and-solutions/mr/nmr-software/topspin.html
NMRPipe	NIST IBBR	http://www.ibbr.umd.edu/nmrpipe/install.html
Sparky	Sparky	https://www.cgl.ucsf.edu/home/sparky/

**Other**		

TCS-SP8 confocal microscope	Leica	Leica TCS-SP8
Upright Microscopes	Leica	Leica DM6 B
600 MHz nuclear magnetic resonance spectrometer	Bruker	Bruker AvanceIII 600MHz

### Resource availability

#### Lead contact

Further information and requests for resources and reagents should be directed to and will be fulfilled by the lead contact, Hao Jiang (haojiang@scu.edu.cn).

#### Materials availability

All unique/stable reagents and materials reported in this study can be generated using protocols detailed in the STAR methods section, or will be shared by the lead contact upon request.

#### Data and code availability

Data reported in this paper will be shared by the lead contact upon reasonable request.

This paper does not report original code.

Any additional information required to reanalyze the data reported in this paper is available from the lead contact upon request.

### Experimental model and study participant details

HeLa cells and HEK293T cells were cultured in DMEM (Thermo Fisher Scientific) supplemented with 10% (v/v) FBS (Excell). The cells were grown at 37°C in a 5% CO2 humidified chamber. sf-9 cells were cultured in Grace’s Insect Medium (Thermo Fisher Scientific), supplemented with 10% (v/v) FBS (Gibco). sf-9 cells were grown at 27°C. All the stably expression cells lines were sustained in medium contained Puromycin (200 ng/μL).

### Method details

#### Constructs

All plasmids mentioned were constructed through homologous recombination by using Uni Seamless Cloning and Assembly Kit (Transgene, CU101). To express 6×His-tagged fusion protein in bacteria, the cDNA encoding hAurora-A, hAurora-IDR (1-126aa), hAurora-ΔIDR (127-403aa) or hAurora-CD (122-403aa) was cloned into pET-28a at BamHI site. The sequence of hAurora-A-11A and hAurora-A-11D were synthesized by Chengdu YouKangjianxing Biotechnology, followed by being cloned into pET-28a vector at BamHI site. The mutations, pET-28a-hAurora-A-R46D, pET-28a-hAurora-A-D274A, pET-28a-hAurora-A-T288A, pET-28a-hAurora-A-S342A and pET-28a-hAurora-A-R195A, were generated on the basis of pET-28a-Aurora-A by using Q5 Site-Directed Mutagenesis Kit (NEB, E0554S) as manual instruction. To create FUNS-11A and FUSN-R46D, the cDNA encode N-terminus of FUS, 11A and R46D was amplified with overlapping segment and co-cloned into pET-28a vector at BamHI site.To create GST-tagged BuGZ-N for pull down assays, the sequence of hBuGZ-N-terminus (1-92aa) was cloned into pGEX-6P-1 vector at EcoRI site.

For overexpression in sf-9 cells, the cDNA of hAurora-A was cloned into pFastBacHTa-mCherry vector at XBaI site.

For overexpression in mammalian cells, the cDNAs of hAurora-A, hAurora-ΔIDR, hAurora-A-11A, hAurora-A-11D, hAurora-A-R46D, hAurora-A-D274A, hAurora-A-T288A and hAurora-A-S342A were cloned to pEGFP-C1 vector at SalI site or pLV-AcGFP-C1 vector at BamHI site. To create GFP-FUNS-11A or GFP-FUSN-R46D, the cDNA encode N-terminus of FUS, 11A and R46D was amplified with overlapping segment and co-cloned into pLV-AcGFP-C1 vector at BamHI site. To create mCherry-H2B used in live-cell imaging, the cDNA encodes H2B was cloned to pLV-mCherry-C1 vector at BamHI site. To produce flag-hAurora-A or flag-hAurora-A-R195A, flag-hAurora-A-2A and flag-hAurora-A-3A constructs for co-immunoprecipitation, the sequence encoding hAurora-A was first cloned into pcDNA3.1-flag vector at SalI site. Flag-hAurora-A-R195A, flag-hAurora-A-2A and flag-hAurora-A-3A were mutated on the basis of flag-hAurora-A at correspondingsites by using Q5 Site-Directed Mutagenesis Kit. To create GFP-tagged BuGZ-N for co-immunoprecipitation, the sequence of BuGZ-N-terminus was cloned into pEGFP-C1 vector at SalI site. To create RFP-hBuGZ or RFP-hBuGZ-ΔN (93-464aa) to assess BuGZ promotes LLPS of Aurora-A in mitosis, corresponding cDNA (isoform 2) was cloned into pRFP-C1 vector at BglII and SalI sites. To create RNAi-insensitive clones, the wobble codons corresponding to the siRNA oligos of Aurora-A were mutated using site-directed mutagenesis. For the detailed information on plasmids construction see Table S1.

#### Protein expression, purification

For expression of His-hAurora-A, His-hAurora-A-IDR, His-hAurora-A-ΔIDR, His-hAurora-A-11A, His-hAurora-A-11D, His-hAurora-A-R46D, His-Aurora-A-FUSN-11A, His-Aurora-A-FUSN-R46D,His-hAurora-A-T288A, His-hAurora-A-D274A, His-hAurora-A-S342A, His-hAurora-A-R195A, His-hAurora-A-CD, and GST-BuGZ-N-terminus, plasmids were transformed into Rosetta (DE3). Protein purification has been clearly described previously.^45^ For expressing His-mCherry-Aurora-A and His-GFP-mBuGZ by using Bac-to-Bac Baculovirus Expression System, plasmids were transformed into DH10Bac to acquire bacmids and expressed in sf-9 cells as the protocol described by Jiang et al.^45^ Further, the P3 virus generated from sf-9 cells was added into High Five cells when the density of cells reached 1.0 × 10ˆ6 cells/ml to get higher protein expression. The next steps to purify His-mCherry-Aurora-A and His-GFP-mBuGZ followed the description mentioned in Jiang’s work.^45^

To produce ^13^C,^15^N-labelled Aurora-A-IDR for NMR, pET-28a-Aurora-A-IDR was transformed into BL21(DE3). The single colony of *E.coli* was cultured in LR medium containing ^15^N-labeled NH_4_Cl (0.5 g/L) and ^13^C-labeled glucose (2.5 g/L). Expression was induced by 0.5 mM IPTG at 16°C for 24 h and purification protocol was the same as above. His tag was removed by TEV cleavage. Then the protein was further purified protein by HPLC. The fraction was lyophilized, redissolved, and dialyzed against NMR buffer (20 mM NaH_2_PO_4_ 50 mM NaCl, 2 mM EDTA, 1 mM DTT). All purified proteins were analyzed and confirmed with SDS-PAGE.

#### Droplets assay and FRAP *in vitro*

10 μL of hAurora-A wild-type or mutants protein at the indicated concentrations were prepared by diluting the stock solutions into prechilled PBS. PEG 6000 was added last to a final concentration of 10% (v/v). The mixture was loading into a flow cell made with two pieces of double sticky scotch taps and the whole device was heated to 37°C for 2 min followed by imaging immediately by using differential interference contrast (DIC) microscope (Leica DM6 B). FRAP assay in cells were performed on inverted confocal microscope (Zeiss LSM 880) with 63×oil objective. Two areas of centrosomal GFP-Aurora-A were bleached simultaneously by using a laser intensity of 95% at 488 nm (for GFP). *In vitro* FRAP experiments for His-mCherry droplets were performed on a structure illumination microscope (Nikon) with a 100×oil objective, while for his-mCherry-Aurora-A on GFP-Aurora-A beads surface, the assay was performed on inverted confocal microscope (Leica SP8) with 63× oil objective. His-mCherry-Aurora-A were bleached using a laser intensity of 50% at 568 nm (for mCherry). Recovery was recorded for the indicated time. The recovery rate was calculated by the final recovery percentage divived by the corresponding time.

#### NMR spectroscopy

^15^N,^13^C-labelled wild-type AuroraA-IDR for backbone assignment was concentrated to 0.5 mM in 20 mM NaH_2_PO_4_ 50 mM NaCl, 2 mM EDTA, 1 mM DTT, 5%D_2_O, pH6.4. IDR titrated with AuroraA Kinase was concentrated to 0.1 mM.

All NMR experiments were performed on Bruker Avance III 600 MHz spectrometerat 278K. The backbone assignments were achieved with standard multidimensional NMR methods as HNCACB, CBCA(CO)NH, HN(CA)CO, HNCO, and (H)N(COCA)NH. All NMR data were processed in Topspin 4 (Bruker), NMRpipe, and analyzed with Sparky and FLYA.

The combined chemical shifts perturbation in ^1^H and ^15^N dimensions were combined as $\sqrt{{\left(\Delta N\times 0.15\right)}^{2}+{\Delta H}^{2}}$. The intensities ratios were defined as I_b_/I_0_, where I_b_ is the peak intensity of a particular residue in the presence of the ligand and I_0_ is the intensity of the same residue when the ligand is absent. The error of I_b_/I_0_ was calculated according to Gauss’ error propagation:

$$\sigma =\frac{I_{b}}{I_{0}}*\sqrt{{\left(\frac{1}{SNR_{b}}\right)}^{2}+{\left(\frac{1}{SNR_{0}}\right)}^{2}}$$

SNR_b_ and SNR_0_ are signal-noise-ratio (SNR) of the peaks in the presence and absence of ligands, respectively.

#### Cell manipulations

For Aurora-A knockdown, we used Aurora-A siRNA oligonucleotide previously published (5′-AUG CCC UGU CUU ACU GUC A-3′),^4^ control oligonucleotide was purchased from Shanghai Genepharma. In brief, 50 pM of each siRNA oligonucleotide was transfected into cells using Lipofectamine RNAiMAX (Invitrogen). To investigate the localization of GFP-Aurora-A-D274A, GFP-Aurora-A-T288A and GFP-Aurora-A-S342A, corresponding plasmids were transfected into HeLa cells by using Lipo 3000 (Invitrogene) for 48 h expression followed by being synchronized to metaphase by 1.5 h treatment of MG132 (10 μM final, MCE, HY-13259). RNAi-insensitive GFP-Aurora-A, GFP-Aurora-A-ΔIDR, GFP-Aurora-A-11A, GFP-Aurora-A-11D, GFP-Aurora-A-R46D, GFP-Aurora-A-FUSN-11A and GFP-Aurora-A-FUSN-R46D stable HeLa cells were used for observation of the localization of Aurora-A-WT or -mutants and rescue assays. GFP-Aurora-A or GFP-Aurora-A-mutants stable HeLa cell lines were derived from HeLa cells by infecting with corresponding lenti-virus for 24 h and followed by selecting with 500 ng/μL Puromycin and sustained in 200 ng/μL Puromycin-contained medium. The stably cell lines for live-cell imaging were derived from HeLa cells by co-infecting with mCherry-H2B and corresponding GFP-Aurora-A lenti-virus for 24 h and followed by selection described above.

Two ways were used for HeLa cell synchronization. The first, cells were first treated with 2.5 mM thymidine (Sigma, T0895) for 20 h and followed by washing with PBS. After releasing into fresh medium for 8 h, the cells were incubated in 2.5 mM thymidine for another 16 h and release. siRNA treatment was executed in the interval of the two thymidine-blocks. The second method, cells were treated with RNAi for 24 h first, then Ro-3306 (10 μM final,MCE, HY-12529) was added to block cells at G2/M boundary and followed by releasing to Ro-3306-free medium. To synchronize cells to prophase, cells were fixed after ∼30 min release. To synchronizing cells to metaphase to assess chromosome alignment, 10 μM MG132 was used to treat cells for 1.5 h. Cells were fixed at indicated time points with 4% PFA followed by immunofluorescence. For live-cell imaging, cells were first synchronized to G2/M boundary by Ro-3306 block for 24 h and released for 30 min to enter mitosis. The cells with broken nucleus envelope, condensed but not aligned chromosomes were recognized as in prometaphase for further imaging, followed by live photograph in the presence of 10 μM MG132.

#### Microtubules regrowth assay

To display regrowth assay in, HeLa cells were first synchronized to G2/M boundary by Ro-3306 and released to DMEM medium for 30 min, then was incubated on ice for 1 h to depolymerize MTs, followed by incubating with warm medium for 1.5 min at 37°C to repolymerize MTs. Cells were fixed by pre-cold methanol in −20°C for 30 min followed by immunofluorescence.

#### Immunofluorescence, microscopy, 3D reconstructuion and live-cell imageing

Immunofluorescence was performed by combining the methods described in previous researches.^10^^,^^45^ Briefly, the fixed cells were permeated by 0.5% PBST (Triton X-100) and blocked in 1% BSA, followed by incubating with first and second antibodies. A DM6 microscope (Leica Microsystems) or a TCS SP8 laser confocal microscope (Leica Microsystems) was used for imaging. For confocal microscopy, 63×, 1.4 NA oil objective lenses (Leica Microsystems) was used, and the cells were imaged by scanning optical sections at ∼0.5-μm intervals. 3D reconstruction was according to confocal microscopy which scaned cell from the top to the bottom of cells based on DAPI fluorescence at ∼0.3-μm intervals, and reconstructed by LAS X software. For live-cell imaging, cells stably expressed mCherry-H2B were plated on a glass-bottom dish and first synchronized to prometaphase followed by 1 h of live photograph. Images were acquired by TCS SP8 laser confocal microscope (Leica Microsystems).

#### Kinase assay *in vitro*

To test whether LLPS of Aurora-A enhanced the kinase activity of Aurora-A, 0.1 μg of His-Aurora-A-WT, His-Aurora-A-11A, His-Aurora-A-R46D, His-Aurora-A-FUSN-11A and His-Aurora-A-FUSN-R46D was used for kinase assay, and 1 μg of His-PLK1 was used as substrate. The protein mixture was incubated in phosphorylation buffer containing 20 mM HEPES, pH 7.5, 10 mM MgCl_2_, 5 mM EGTA, 1 mM DTT, 1 μM ATP in a total volume of 30 μL. The kinase reaction was performed at 30°C for 30 min and was terminated by adding 10 μL of 4×SDS sample buffer and boiled for 10 min. The sample was followed by Western Blot and analyzed by phosphorylation antibody of PLK1 (pT210, BD) to assess kinase activity of Aurora-A.

#### Molecular docking

The molecular docking was entrusted to Wuhan Yangene Biological Technology. Simply, the molecular docking was performed based on the structure of Aurora-A catalytic (PDB ID: 1MQ4). The BuGZ-N sequences were obtained from the Uniprot database. After selecting the target sequence, the initial simulation structure of BuGZ-N is obtained by the I-TASSER online server (https://zhanggroup.org/I-TASSER/) through ab initio modeling, and then is energy-optimized by the Rosetta Relax module to obtain the final structure. Flexible docking was performed using the RosettaDock. The lowest energy conformation was selected for molecular dynamics simulation. The protein docking binding energy was −23.727 kcal/mol. Molecular dynamics simulation (MD) was conducted using the GROMACS 2020.6 package (https://manual.gromacs.org/) with the Charmm36 force field and TIP3P explicit water model, and the system was electrically neutral by adding counterions. Subsequently, energy minimization (1000.0 kJ/mol/nm) was performed using the steepest descent method. Canonical ensemble simulation (NVT, 100 ps) and isothermal isobaric simulation (NPT, 100 ps) were used to guarantee that the system could endure constant temperature and pressure (310 K, 1 bar). The annealing method was used to ensure that the system was slowly warmed from 0 to 310 K within 150 ps in the NVT simulation process. Finally, a 30-ns MD simulation was started, and the GPU processor was used to accelerate MD simulations. After 30 ns of molecular dynamics, the RMSD values reach equilibrium and the output protein complex stabilizes the trajectory conformation.

#### Interaction analyses

To verify the interaction of hAurora-A and hBuGZ, we performed pull down assay. 5 μg of purified His-hAurora-A-CD (catalytic domain) and 5 μg of GST-BuGZ-N (N-terminus) or GST were incubated with glutathione-agarose (Thermo Fisher Scientific, #16101) in 200 μL 0.1% PBST (Triton X-100) at 4°C for 2 h. After washing with 0.5% PBST and PBS for three times respectively, the sample was resuspended in SDS-sample to a total volume of 100 μL and boiled for 10 min followed by SDS-PAGE and Western Blot analysis. 0.1% of total reaction and 20% of total pulldowns were analyzed by Western blotting. 1% of total reaction and 10% of total pulldowns were analyzed by Coomassie blue staining.

To further confirm the interaction of Aurora-A and BuGZ based on the result of molecular docking, flag-luciferase, flag-Aurora-A-WT or flag-Aurora-A-mutants (flag-Aurora-A-R195A, flag-Aurora-A-2A and flag-Aurora-A-3A) was co-expressed with GFP-BuGZ-N in HEK293T cells for 24 h to perform co-immunoprecipitations. Cells were collected by using lysis buffer (Beyotime, P0013) contained PMSF (Roche, 329-98-6) and EDTA-free protease inhibitor cocktail (MCE, HY-K0010), and the mixture was followed by centrifugation at 10,000 rpm for 20 min at 4°C. The cell lysate was incubated with 10 μL flag-agarose (sigma, A2220) for 2 h at 4°C. After washing with 0.5% PBST (Triton X-100) and PBS for three times respectively, the agarose was resuspended in SDS sample buffer to a total volume of 100 μL and boiled for 10 min. For Western blotting analyses, 1% of input lysate and 10% of immunoprecipitates were loaded.

Mitotic HeLa cells stably expressed GFP-Aurora-A were used to perform reciprocal immunoprecipitations to determine the different interaction of Aurora-A and BuGZ during G2 phase and mitosis. HeLa cells in G2 phase were collected after 16 h of Ro-3306 treatment, while mitotic HeLa cells were acquired 1.5 h after Ro-3306 release in the presence of MG132. The next steps were followed above with substituting flag-beads with GFP-Trap agarose (Chromotek, gta-20).

#### Sequence analysis for protein disorder

The PONDR program (http://www.pondr.com/) was used to analyze disordered regions of Aurora-A. Three indicators (VLS2, VL-XT, and XL1-XT) showed Aurora-A possesses intrinsic disordered region.

### Quantification and statistical analysis

The definitions and exact values of n, distributions and deviations form experiments are presented in the corresponding Figure Legends. Unless stated otherwise within the text, statistical analysis was performed using GraphPad Prism. Error bars for all data represent SEM. Statistical significance was determined using two-tailed *Student*’s t-test, n.s., no significance, ^∗∗^*p* < 0.01, ^∗∗∗^*p* < 0.001, ^∗∗∗∗^*p* < 0.0001.

#### Western blot intensity statistics

The western blot intensity was measured and calculated by ImageJ.

#### Fluorescence intensity statistics

For centrosomal γ-tubulin, α-tubulin or GFP intensity measurement, the area of interest was defined by drawing a circle including the centrosome and background value is measured from the same-sized circle as a circle including the centrosome in an adjacent region.^10^ To estimate spindle intensity, two circle with area of ∼200 μm^2^ regions, corresponding to the brightest areas of each half spindle was chosen based on tubulin intensity and the average intensities in these areas were determined. The background fluorescence was subtracted using the intensity measured in areas away from the spindle.^45^ Fluorescence intensity was analyzed based on z stacks acquired with confocal microscopy. It was calculated by using LAS X software. The methods used for this study were as following: Integrated density = (Integrated density of ROI - Integrated density of background region/Area of background region × Area of ROI)/Area of ROI.

#### Software availability

The ImageJ used for western blot intensity analysis is available at https://imagej.nih.gov/ij/. Prism 8.0 (GraphPad) is used in this study and is available at https://www.graphpad.com/.

## Supplemental information

Document S1. Figures S1–S4

Table S1. Primers used for constructs, related to the STAR Methods, “Constructs” section
