# Supplementary material for: Aurora-A condensation mediated by BuGZ aids its mitotic centrosome functions
Source: iScience. 2024 Apr 18;27(5):109785. doi: 10.1016/j.isci.2024.109785 (PMC11090908; doi:10.1016/j.isci.2024.109785)
Supplement: Document S1. Figures S1–S4 [file mmc1.pdf]

iScience, Volume *27*

Supplemental information

Aurora-A condensation mediated

by BuGZ aids its mitotic centrosome functions

Hui Zheng, Qiaoqiao Zhang, Xing Liu, Fan Shi, Fengrui Yang, Shengqi Xiang, and Hao Jiang

Figure S1

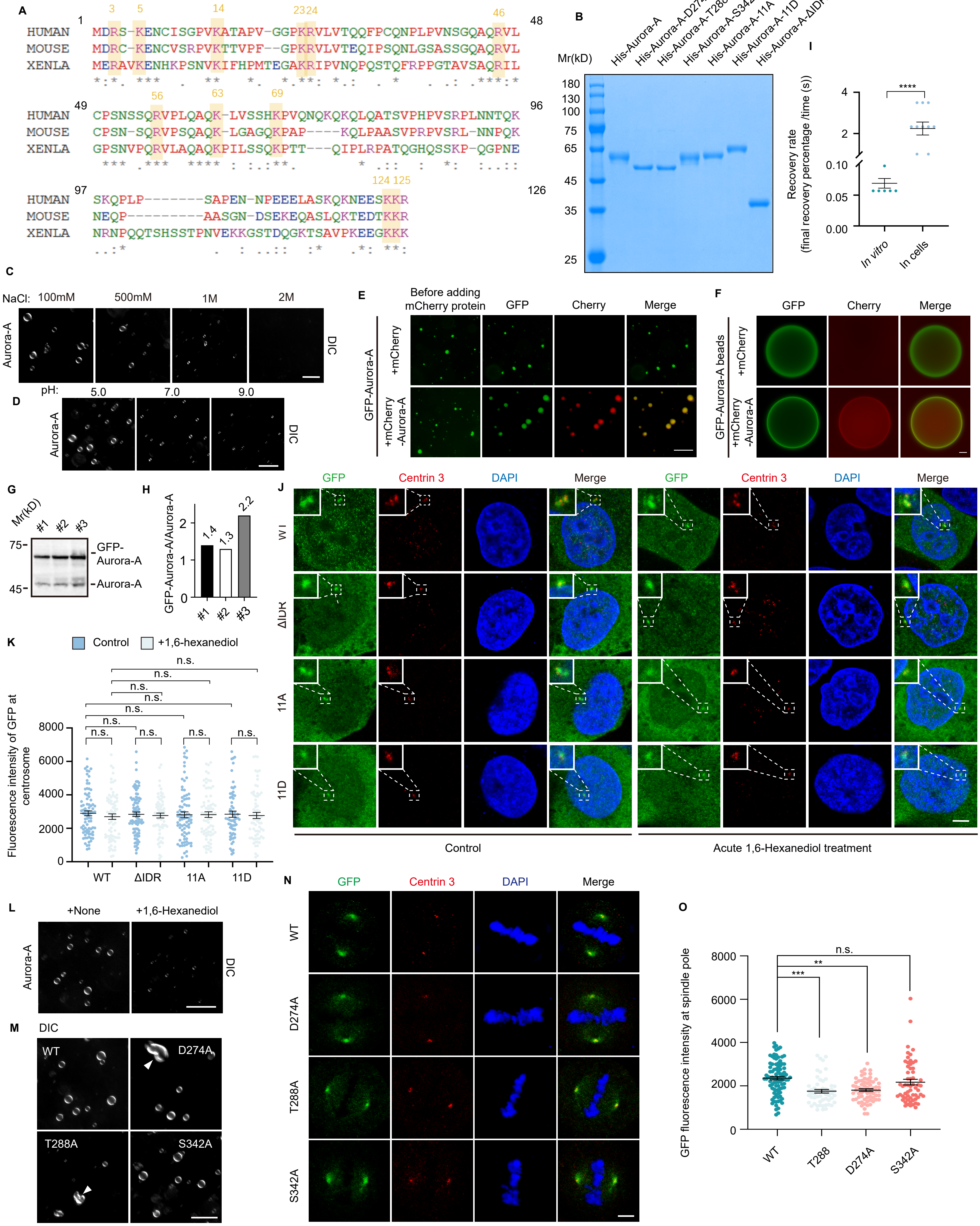
A
HUMAN MDRS-KENCISGPVKATAPV-GGPKRVLVTQQFPCQNPLPVNSGQAQRVL
MOUSE MDRC-KENCVSRPVKTTVPF--GPKRVLVTEQIPSQNLGSASSGQAQRVL
XENLA MERAVKENHKPSNVKIFHPMTEGAKRIPVNQPQSTQFRPPGTAVSAQRIL
HUMAN CPSNSSQRVPLQAQK-LVSSHKPVQNQKQKQLQATSVPHPVSRPLNNTQK
MOUSE CPSN-SQRVPSQAQK-LGAGQKPAP----KQLPAASVPRPVSRL-NNPQK
XENLA GPSNVPQRVLAQAQKPILSSQKPTT---QIPLRPATQGHQSSKP-QGPNE
HUMAN SKQPLP-------SAPEN-NPEEELASKQKNEESKKR
MOUSE NEQP---------AASGN-DSEKEQASLQKTEDTKKR
XENLA NRNPQQTSHSSTPNVEKKGSTDQGKTSAVPKEEGKKK
B
Mr(kD)
His-Aurora-A
His-Aurora-A-D274A
His-Aurora-A-T288A
His-Aurora-A-S342A
His-Aurora-A-11A
His-Aurora-A-11D
His-Aurora-A-ΔIDR
I
Recovery rate (final recovery percentage /time (s))
In vitro
In cells
C
NaCl: 100mM 500mM 1M 2M
Aurora-A
DIC
D
pH: 5.0 7.0 9.0
E
Before adding mCherry protein
GFP
Cherry
Merge
GFP-Aurora-A
+mCherry
+mCherry -Aurora-A
F
GFP-Aurora-A beads
G
Mr(kD)
GFP-Aurora-A
Aurora-A
H
GFP-Aurora-A/Aurora-A
J
Centrin 3
DAPI
WT
ΔIDR
11A
11D
Control
Acute 1,6-Hexanediol treatment
K
Control
+1,6-hexanediol
n.s.
Fluorescence intensity of GFP at centrosome
L
+None
+1,6-Hexanediol
M
DIC
D274A
T288A
S342A
N
O
GFP fluorescence intensity at spindle pole
T288

Figure S2

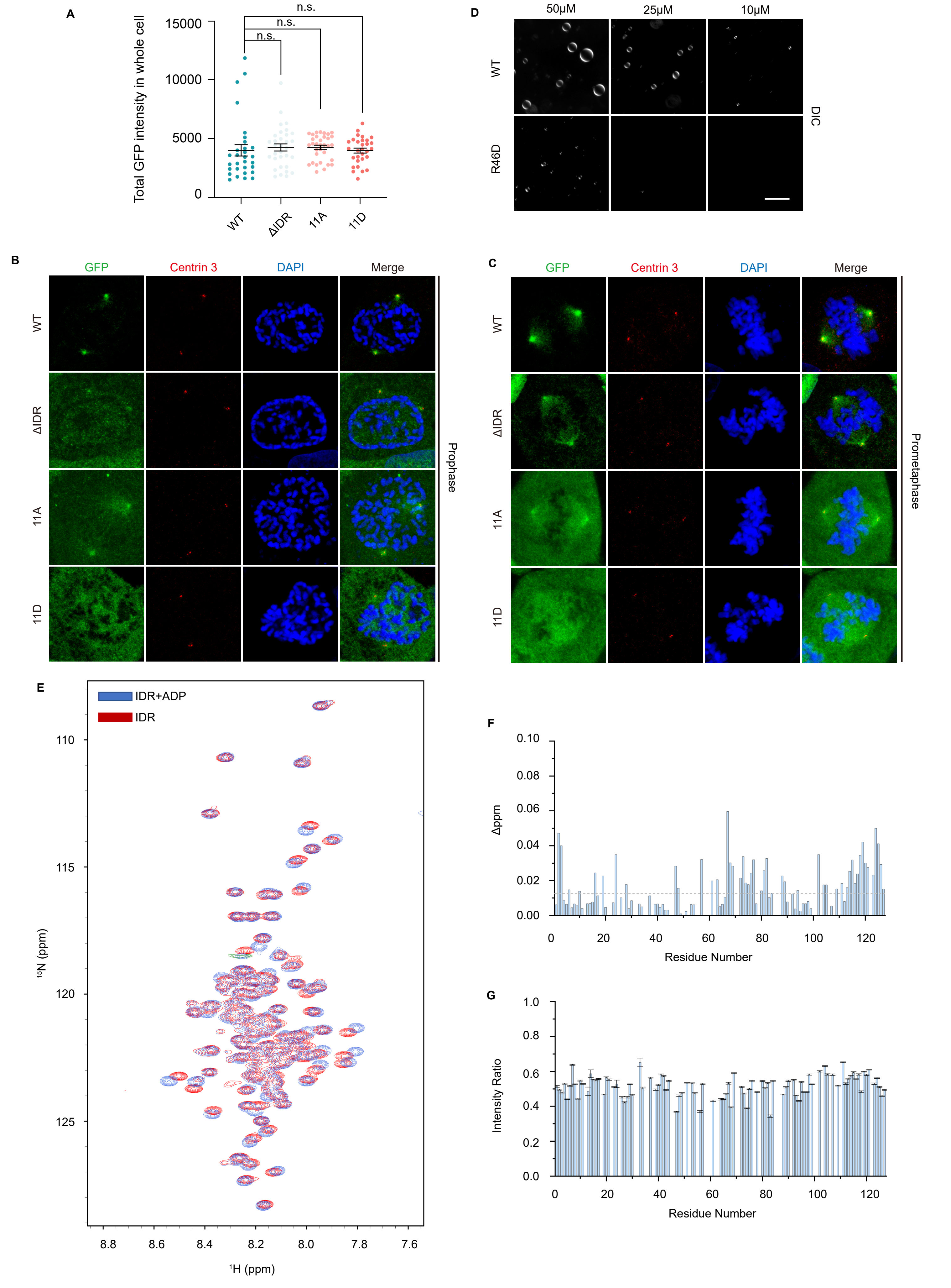

A
Total GFP intensity in whole cell
15000
10000
5000
0
n.s.
n.s.
n.s.
WT
ΔIDR
11A
11D
D
50μM
25μM
10μM
WT
R46D
DIC
B
GFP
Centrin 3
DAPI
Merge
WT
ΔIDR
11A
11D
Prophase
C
GFP
Centrin 3
DAPI
Merge
WT
ΔIDR
11A
11D
Prometaphase
E
IDR+ADP
IDR
110
115
120
125
15N (ppm)
8.8
8.6
8.4
8.2
8.0
7.8
7.6
1H (ppm)
F
0.10
0.08
0.06
0.04
0.02
0.00
Δppm
0
20
40
60
80
100
120
Residue Number
G
1.0
0.8
0.6
0.4
0.2
0.0
Intensity Ratio
0
20
40
60
80
100
120
Residue Number

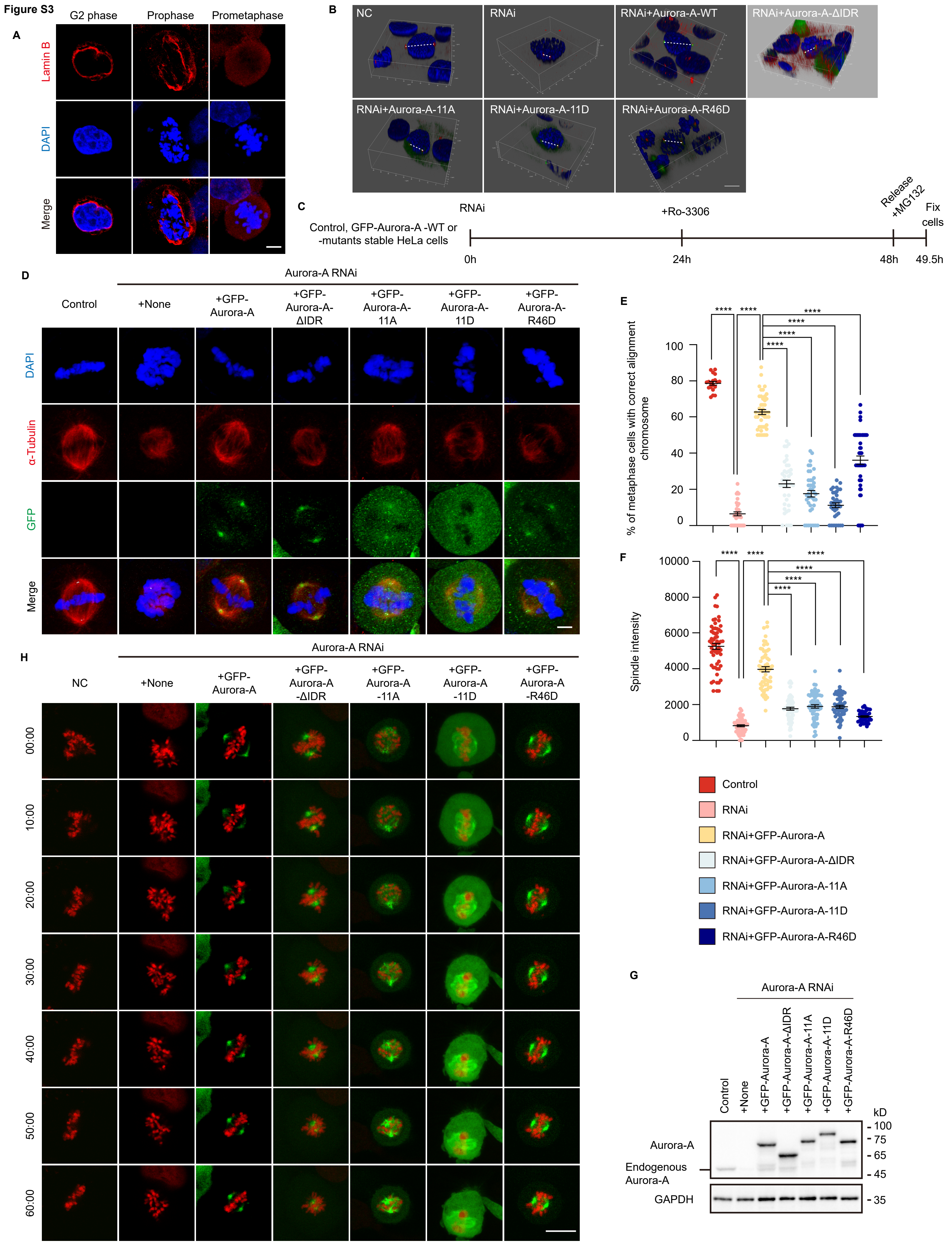
Figure S3
A
G2 phase
Prophase
Prometaphase
Lamin B
DAPI
Merge
B
NC
RNAi
RNAi+Aurora-A-WT
RNAi+Aurora-A-ΔIDR
RNAi+Aurora-A-11A
RNAi+Aurora-A-11D
RNAi+Aurora-A-R46D
C
RNAi
+Ro-3306
Release +MG132
Fix cells
Control, GFP-Aurora-A -WT or -mutants stable HeLa cells
0h
24h
48h
49.5h
D
Aurora-A RNAi
Control
+None
+GFP-Aurora-A
+GFP-Aurora-A-ΔIDR
+GFP-Aurora-A-11A
+GFP-Aurora-A-11D
+GFP-Aurora-A-R46D
DAPI
α-Tubulin
GFP
Merge
E
% of metaphase cells with correct alignment chromosome
****
F
Spindle intensity
Control
RNAi
RNAi+GFP-Aurora-A
RNAi+GFP-Aurora-A-ΔIDR
RNAi+GFP-Aurora-A-11A
RNAi+GFP-Aurora-A-11D
RNAi+GFP-Aurora-A-R46D
G
Aurora-A RNAi
Control
+None
+GFP-Aurora-A
+GFP-Aurora-A-ΔIDR
+GFP-Aurora-A-11A
+GFP-Aurora-A-11D
+GFP-Aurora-A-R46D
kD
Aurora-A
Endogenous Aurora-A
GAPDH
H
Aurora-A RNAi
NC
+None
+GFP-Aurora-A
+GFP-Aurora-A-ΔIDR
+GFP-Aurora-A-11A
+GFP-Aurora-A-11D
+GFP-Aurora-A-R46D
00:00
10:00
20:00
30:00
40:00
50:00
60:00

Figure S4

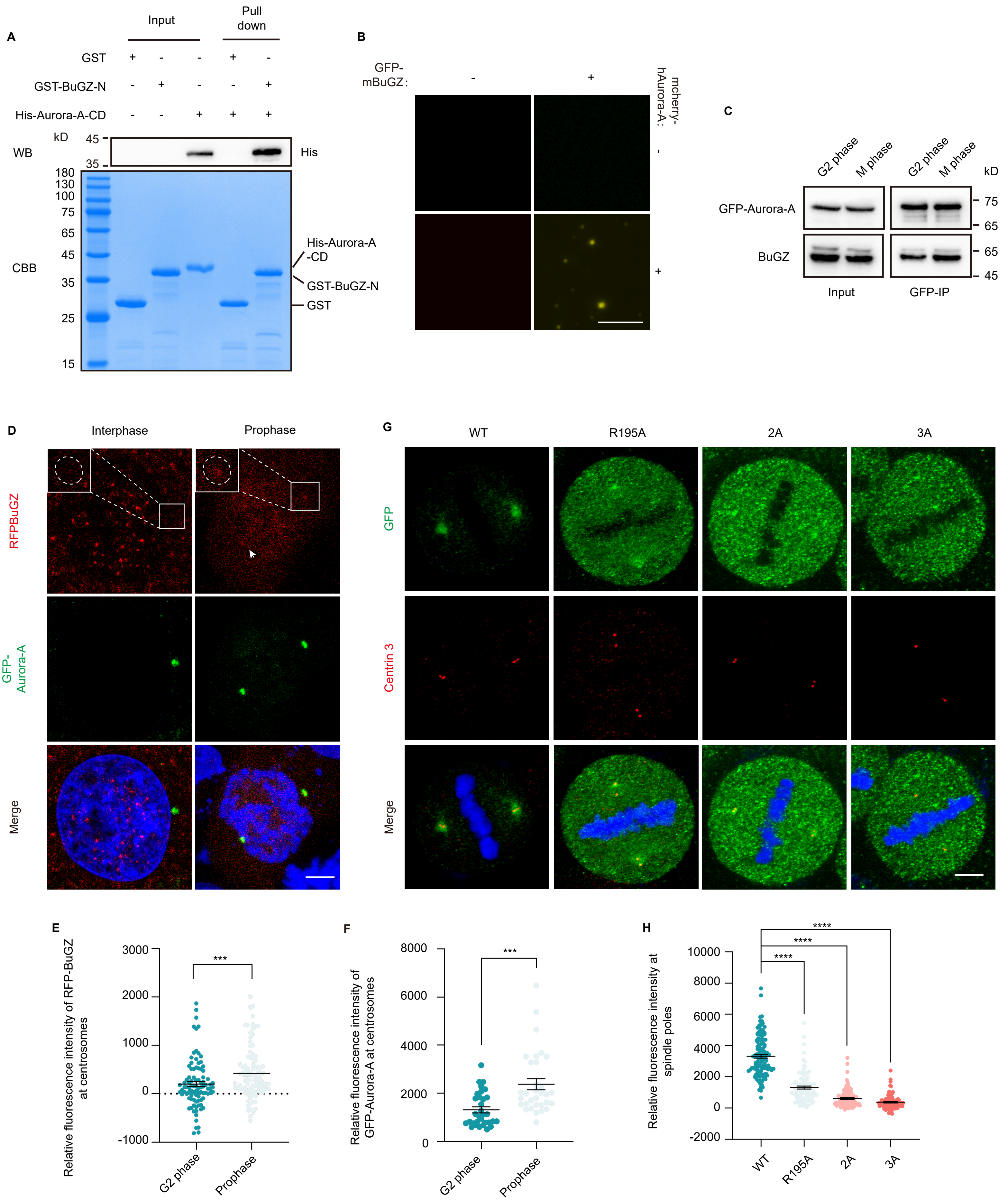

A
Input
Pull down
GST
GST-BuGZ-N
His-Aurora-A-CD
kD
45
35
WB
His
180
130
100
75
65
45
35
25
15
CBB
His-Aurora-A -CD
GST-BuGZ-N
GST
B
GFP-mBuGZ:
mcherry-hAurora-A:
C
G2 phase
M phase
kD
GFP-Aurora-A
BuGZ
75
65
65
45
Input
GFP-IP
D
Interphase
Prophase
RFPBuGZ
GFP-Aurora-A
Merge
G
WT
R195A
2A
3A
GFP
Centrin 3
Merge
E
Relative fluorescence intensity of RFP-BuGZ at centrosomes
3000
2000
1000
0
-1000
***
G2 phase
Prophase
F
Relative fluorescence intensity of GFP-Aurora-A at centrosomes
8000
6000
4000
2000
0
***
G2 phase
Prophase
H
Relative fluorescence intensity at spindle poles
10000
8000
6000
4000
2000
0
-2000
****
****
****
WT
R195A
2A
3A

Figure S1. Aurora-A undergoes LLPS *in vitro* but not in interphase centrosomes, related to Figure 1.

(A) Sequence comparisons between hAurora-A, mAurora-A and xAurora-A. The orange shading box indicates conserved positively charged residues in the IDR domain.

(B) Coomassie brilliant blue staining of purified His-Aurora-A-WT, -D274A, -T288A, -S342A, -11A, -11D and -ΔIDR proteins.

(C) Images of droplets of His-Aurora-A (50μM) at different ionic concentrations. Scale bar, 10μm.

(D) Images of droplets of His-Aurora-A (50μM) at different pH values concentrations. Scale bar, 10μm.

(E) Images of His-GFP-Aurora-A (25μM) droplets with adding the mCherry-tagged protein (25μM). Scale bar, 5μm.

(F) Images of GFP-Aurora coated beads with adding the purified mCherry-tagged proteins (10μM). GFP-Aurora-A coated beads were produced by pre-incubating GFP-beads (10μL) with GFP-Aurora-A stable expressed HeLa cells (1×10^7 cells) lysate. Scale bar, 5μm.

(G) The endogenous and exogenous protein levels in three of GFP-Aurora-A stably expressed cell lines were indicated by Western blotting.

(H) Ratio of GFP-Aurora-A intensity/endogenous Aurora-A intensity corresponding to (G) was measured by Image J. The cell line #2 was used for FRAP assay and 1,6-hexanediol treatment.

(I) Comparation of recovery rate between *in vitro* and in cell corresponding to FRAP experiment. 7 of *in vitro* data and 11 of *in vivo* data were calculated.

(J-K) Images (J) and quantification (K) of centrosomal localization of GFP-Aurora-A, GFP-Aurora-A-ΔIDR, GFP-Aurora-A-11A and GFP-Aurora-A-11D with or without 1,6- hexanediol treatment in interphase cells. 50-55 total cells from three independent experiments in each experiment were measured and quantified. Scale bar, 5μm.

(L) Images of droplets of His-Aurora-A (40μM) with or without 10% 1,6-Hexaneidol treatment *in vitro* as visualized by DIC microscopy. Scale bar, 10μm.

(M) Images of His-Aurora-A-WT, His-Aurora-A-D274A, His-Aurora-A-T288A, and His-Aurora-A-S342A droplet formation *in vitro* as visualized by DIC microscopy. The white arrows indicate incomplete fusion. Protein concentration, 50μM. Scale bar, 10μm.

(N-O) Images (N) and quantification (O) of centrosomal localization of GFP-Aurora-A-WT, GFP-Aurora-A-D274A, GFP-Aurora-A-T288A, and GFP-Aurora-A-S342A in metaphase cells. 45-50 total cells from three independent experiments in each experiment were measured and quantified. Scale bar, 5μm.

Error bars indicate SEM. *Student's t* test: n.s., no significance; **, P < 0. 01; ***, P < 0.001; ****, P < 0.0001.

Figure S2. LLPS of Aurora-A occurs in mitotic centrosome and is mediated by site-specific intramolecular interactions, and ADP binding to the IDR region

might also modulate the LLPS behavior of Aurora-A, related to Figure 2.

(A) Quantification of total GFP intensity of whole cell. 30-33 total cells from three independent experiments in each experiment were quantified.

(B-C) Images of centrosomal localization of GFP-Aurora-A-WT, GFP-Aurora-A-ΔIDR, GFP-Aurora-A-11A and GFP-Aurora-A-11D in prophase (B) and prometaphase (C) cells. Centrin 3 marks centrosomes. Scale bar, 5μm.

(D) Droplets formation of His-Aurora-A-WT and His-Aurora-A-R46D *in vitro*, as visualized by DIC microscopy. Protein concentration was from 10 to 50μM. Scale bar, 10μm.

(E) Overlay of 2D ^{1}H-^{15}N HSQC measured at 278K, in the absence (red) and the presence (blue) of ADP (20mM).

(F) The chemical shift perturbations of IDR induced by 20mM ADP

(G) The peak intensities changes of IDR induced by 20mM ADP.

Figure S3. LLPS of Aurora-A contributes to centrosomes separation, spindle formation and chromosome alignment, related to Figure 4.

(A) Images of Lamin B staining of HeLa cells in G2 phase, prophase and prophase. Scale bar, 5μm.

(B) Images of 3D reconstruction. Dash line indicated the inter-centrosome distance. Red, centrin 3; blue, DAPI. Scale bar, 10μm.

(C) Scheme of cell synchronization and RNAi treatment corresponding to (D) and (H).

(D-G) Cells manipulated by RNAi and rescue treatment were stained with DAPI to assess chromosome alignment in metaphase and with α-tubulin to monitor spindle intensity (D). The percentage of correctly aligned chromosomes (E) and relative fluorescence intensity of spindle α-tubulin (F) were measured. 500-1000 toral cells from three independent experiments in each experiment were counted to assess chromosome misalignment, and 42-50 total cells from three independent experiments in each experiment were measured and quantified for spindle intensity. The endogenous and exogenous protein levels were indicated by Western blotting (G). Scale bar, 5μm.

(H) Images of live cell imaging. Scale bar, 5μm.

Error bars indicate SEM. *Student's t test*: ****, $P < 0.0001$.

Figure S4. BuGZ facilitates Aurora-A condensate *in vitro* and in mitotic centrosomes by directly binding to Aurora-A, related to Figure 5.

(A) Purified GST, GST-BuGZ-N were incubated with His-Aurora-A-CD followed by GST pulldown, Western blotting, and Coomassie blue staining.

(B) Images of co-separation of His-mCherry-Aurora-A (5μM) and His-GFP-mBuGZ (1μM), visualized by fluorescence microscopy. Scale bar, 10μm.

(C) Co-immunoprecipitation of GFP-Aurora-A and endogenous BuGZ by using G2 phase or mitotic GFP-Aurora-A stable cells lysate.

(D-F) Images (D) and quantification of RFP-BuGZ (E) or GFP-Aurora-A (F) in G2 or mitotic centrosomes. 47-50 total cells from three independent experiments in each experiment were measured and quantified. Scale bar, 5μm.

(G-H) Images (G) and quantification (H) of centrosomal localization of GFP-Aurora-A-WT, GFP-Aurora-A-R195A, GFP-Aurora-A-2A and GFP-Aurora-A-3A mutants in metaphase cells. Centrin 3 indicates centrosomes, 50-60 total cells from three independent experiments in each experiment were measured and quantified. Scale bar, 5μm.

Error bars indicate SEM. *Student's t test*: ***, $P < 0.001$; ****, $P < 0.0001$.
